# Heterogeneity in leukemia cells that escape drug-induced senescence-like state

**DOI:** 10.1038/s41419-023-06015-4

**Published:** 2023-08-05

**Authors:** David Miller, Kyra Kerkhofs, Farnoosh Abbas-Aghababazadeh, Sahib Singh Madahar, Mark D. Minden, Josée Hébert, Benjamin Haibe-Kains, Mark A. Bayfield, Samuel Benchimol

**Affiliations:** 1 Department of Biology, York University, Toronto, ON M3J 1P3 Canada; 2 Princess Margaret Cancer Centre, University Health Network and University of Toronto, Toronto, Canada; 3 Division of Hematology-Oncology, Maisonneuve-Rosemont Hospital and Department of Medicine, Université de Montréal, Montreal, QC Canada; 4 Medical Biophysics, University of Toronto, Toronto, Canada; 5 Vector Institute for Artificial Intelligence, Toronto, Canada; 6 Ontario Institute for Cancer Research, Toronto, Canada; 7 Department of Computer Science, University of Toronto, Toronto, Canada; 8 Department of Biostatistics, Dalla Lana School of Public Health, Toronto, Canada

**Keywords:** Tumour heterogeneity, Senescence, Acute myeloid leukaemia

## Abstract

Erythropoietin (EPO) suppresses drug-induced apoptosis in EPO-receptor-positive leukemia cells and allows cells to persist after drug treatment by promoting cellular senescence. Importantly a small proportion of senescent cells can re-enter the cell cycle and resume proliferation after drug treatment, resulting in disease recurrence/persistence. Using a single-cell assay to track individual cells that exit a drug-induced senescence-like state, we show that cells exhibit asynchronous exit from a senescent-like state, and display different rates of proliferation. Escaped cells retain sensitivity to drug treatment, but display inter-clonal variability. We also find heterogeneity in gene expression with some of the escaped clones retaining senescence-associated gene expression. Senescent leukemia cells exhibit changes in gene expression that affect metabolism and senescence-associated secretory phenotype (SASP)-related genes. Herein, we generate a senescence gene signature and show that this signature is a prognostic marker of worse overall survival in AML and multiple other cancers. A portion of senescent leukemia cells depend on lysosome activity; chloroquine, an inhibitor of lysosome activity, promotes senolysis of some senescent leukemia cells. Our study indicates that the serious risks associated with the use of erythropoietin-stimulating agents (ESAs) in anemic cancer patients may be attributed to their ability to promote drug-tolerant cancer cells through the senescence program.

## Introduction

Acute myeloid leukemia (AML) is a clonal malignancy arising in myeloid progenitor cells. AML remains the most lethal form of blood cancer, with a mortality of nearly 70% in patients 65 years and older. For a majority of patients who initially respond well, the disease recurs within 3 years or diagnosis or combination chemotherapy [1]; such curative intent, combination chemotherapy, often involves cytarabine and daunorubicin (DNR). Chemotherapy-induced genotoxic damage to cells results either in cell death or cellular senescence.

Senescence has traditionally been defined as a stress-induced, permanent state of cell cycle arrest, where the cells remain metabolically active and are resistant to apoptosis. Senescent cells undergo reprogramming and display a senescent-associated secretory phenotype that can regulate intrinsic and extrinsic pathways and influence tissue homeostasis [2–4]. Many endogenous and environmental stresses can promote senescence; in most cases, cellular senescence depends on the activation of the p53/p21 axis and/or the RB/p16 pathway. Previously, we reported that erythropoietin (EPO) protects murine myeloid leukemia DA3/EPOR cells from p53-mediated apoptosis, and instead promotes a senescent state [5]. Although senescence has been thought to be a favorable outcome of chemotherapy, recent reports suggest that cancer cells are able to escape from therapy-induced senescence. Hence, cellular senescence may allow cancer cells to survive during drug treatment, and escape from senescence may contribute to subsequent tumor cell expansion and patient relapse [6, 7]. Moreover, tumor cells that escape senescence have been shown to acquire a more malignant phenotype [6, 8].

Cancer relapse following chemotherapy is often associated with the acquisition of mutations in a subpopulation of cancer cells that confer drug resistance. As well, alternative epigenetic mechanisms causing drug tolerance are emerging and have clinical significance [9–11]. Mechanisms surrounding non-genetic drug tolerance are largely unknown. Recently, a stress-induced embryonic dormancy state known as embryonic diapause (from here on referred to as diapause) was found to contribute to drug resistance in colon cancer, adenocarcinoma, and AML [6, 12, 13].

EPO and erythropoiesis-stimulating agents (ESAs) have been used to manage chemotherapy-induced anemia in cancer patients [14]. Several studies have reported that treatment of anemic, non-leukemic cancer patients with ESAs increases tumor progression, tumor recurrence, thrombosis, and death [15, 16]. These serious safety concerns have resulted in the restricted use of ESAs in patients receiving chemotherapy.

Here we demonstrate that EPO protects DA3/EPOR leukemia cells from drug-induced cell death by promoting and maintaining senescence. Importantly, drug-induced cellular senescence is reversible in DA3/EPOR. Recovered cells, that in the absence of a drug, escape drug-induced senescence are heterogenous in that they display asynchronous release from senescence, varying rates of proliferation, differential responses to second round drug-treatment, and differences in gene expression. During senescence, DA3/EPOR cells undergo a partially reversible reprogramming, gaining a diapause-like phenotype and increasing expression of coagulation-related genes. Despite inter-clonal heterogeneity, RNA-seq revealed that the bulk drug-free recovered population mirrored the naïve, never drug-treated DA3/EPOR cells. We demonstrate that high expression of the senescent signature is associated with decreased survival in AML and multiple other cancers. We show that human and murine leukemia cells undergoing therapy-induced senescence depend on lysosome function and that inhibition of lysosome function with chloroquine (CQ) promotes senolysis.

## Results

### DA3/EPOR leukemia cells persist drug treatment and enter senescence

**Fig. 1:**
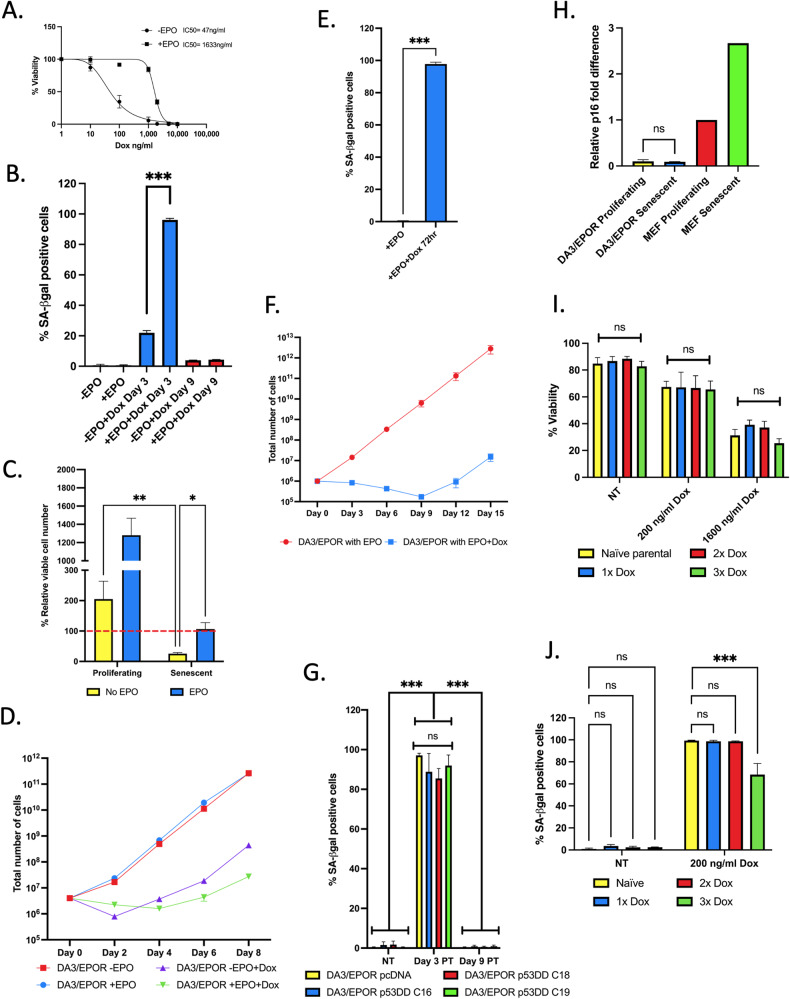
DA3/EPOR cells enter a drug-induced senescent state. **A** Percentage of viable DA3/EPOR cells treated for 72 h with increasing amounts of doxorubicin (Dox) in the presence or absence of erythropoietin (EPO). Error bars represent SEM, *n* = 3 biological replicates; a minimum of 100 cells were counted for each biological replicate. **B** Percentage of senescent DA3/EPOR cells after treatment with Dox for 24 h. SA-β-gal staining of DA3/EPOR cells was performed 3 and 9 days post-treatment. DA3/EPOR cells were treated with or without 200 ng/ml Dox for 24 h in the absence or presence of EPO. After drug treatment, all cells were maintained in complete, EPO-containing media. Related to Supplementary Fig. 1A. Mean ± SEM, *n* = 3 independent experiments; Two-way student *t*-test. A minimum of 100 cells were counted for each independent experiment. **C** Percentage of viable senescent and proliferating DA3/EPOR cells that were maintained in media with or without EPO for 48 h. The proportion of viable cells was normalized to the initial number of cells seeded. Mean ± SEM, n = 4 independent experiments; two-way ANOVA. **D**. Proliferation of DA3/EPOR cells treated with or without 200 ng/ml Dox in the absence or presence of EPO. After 24 h drug treatment, all cells were maintained in complete, EPO containing media. Cells were counted every 2 days. Related to Supplementary Fig. 1A. Mean ± SEM, *n* = 3 independent experiments. Please note that the error bars are small and cannot be visualized on the exponential scale. **E** Percentage of senescent DA3/EPOR cells after treatment with Dox for 72 h. Cells were treated with Dox for 72 h and stained for SA-β-gal 24 h after Dox treatment. Related to Supplementary Fig. 1C. Mean ± SEM, *n* = 3 independent experiments; Two-way Student *t*-test. A minimum of 100 cells were counted for each independent experiment. **F** Proliferation of DA3/EPOR treated with or without 200 ng/ml Dox for 72 h in the presence of EPO. Cells were counted every 3 days. **G** SA-β-gal staining of DA3/EPOR cells expressing p53DD (clones C16, C18, and C19) and empty vector (pcDNA). SA-β-gal staining was performed 3 and 9 days post-treatment of control and Dox-treated cells. Mean ± SEM, *n* = 3 independent experiments; one-way ANOVA. A minimum of 100 cells were counted for each independent experiment. **H** p16 RNA expression in proliferating and senescent DA3/EPOR cells determined by qRT-PCR. Untreated and 3-day post-Dox treated MEFs were included as a positive control for p16 expression. Mean ± SEM, *n* = 3 independent experiments for proliferating and senescent DA3/EPOR cells, *n* = 1 for proliferating and senescent cells MEFs; two-way student *t* test. **I**. Percentage of viable DA3/EPOR and drug-recovered DA3/EPOR cells with increasing concentrations of Dox for 48 h relative to control-treated DA3/EPOR. Drug-recovered DA3/EPOR cells were generated by treating naïve DA3/EPOR cells with 200 ng/ml Dox for 24 h in EPO and allowing the population to recover. Drug-recovered cells that went through 1, 2, and 3 rounds of dox treatment are indicated by 1x Dox, 2x Dox, and 3x Dox, respectively. Cells were stained with trypan blue 48 h drug treatment and cells were counted using a hemocytometer. Mean ± SEM, *n* = 3 independent experiments; two-way ANOVA. **J**. SA-β-gal staining of DA3/EPOR and drug-recovered DA3/EPOR populations performed 3 days and 9 days after treatment with Dox. Mean ± SEM, *n* = 3 independent experiments; two-way ANOVA. A minimum of 100 cells were counted for each independent experiment. Statistical significance is shown by ns, not significantly different, *P < 0.05, **P < 0.01, ***P < 0.001.

To determine whether drug-induced senescence is a reversible phenotype in transformed cells, we employed the DA3/EPOR murine myeloid leukemia cell line [17]. Previously, we demonstrated that EPO protected DA3/EPOR cells from p53-induced death upon exposure to genotoxic and non-genotoxic agents and promoted a senescence-like state [5]. EPO-supplemented DA3/EPOR cells demonstrated a ~35-fold reduced sensitivity to doxorubicin (Dox)-induced cell death compared with cells treated with Dox alone (Fig. 1A). We treated DA3/EPOR cells with Dox in the presence or absence of EPO for 24 h and cultured the cells in EPO without Dox for 3 days. On day 4 (3 days after Dox removal), we observed that 96–99% of surviving DA3/EPOR cells (Dox+/EPO+) and about 20% of drug-treated cells not supplemented with EPO (Dox+/EPO−) entered senescence on the basis of SA-β-gal staining, a well-established bio-marker of cellular senescence (Fig. 1B; Supplementary Fig. 1A–C). To determine if EPO was required to maintain the senescence phenotype, we removed EPO from the senescent cells on day 4 and cultured the cells for 2 additional days. Naïve DA3/EPOR cells in the absence of EPO remained viable, albeit had a reduced rate of proliferation. Withdrawal of EPO from senescent DA3/EPOR cells led to an ~5-fold decrease in cell viability on day 6 indicating that EPO sustains the viability and the senescence program (Fig. 1C). Dox+/EPO+-treated cells exhibited increased cell size and accumulated in the G1 and G2/M phases of the cell cycle (Supplementary Fig. 1B, D, E), two additional features associated with cellular senescence. Next, we measured the proliferative capacity of DA3/EPOR cells treated with Dox in the presence and absence of EPO by counting viable cells every 2 days, over an 8-day course. Dox+/EPO+ cells exhibited a slight decrease in viable cell number for 4 days as expected for a population of primarily senescent cells. An increase in cell number, however, was detected on day 6 and continued into day 8 concomitant with a decrease in the proportion of SA-β-gal positive senescent cells (Fig. 1D; Supplementary Fig. 1C). By 9 days post-dox, the levels of SA-β-gal positivity was comparable to the naïve population (Fig. 1B; Supplementary Fig. 1C). Cells treated with Dox in the absence of EPO showed a marked decrease in cell number consistent with Fig. 1A. Cells that survived drug treatment in the absence of EPO resumed growth after the removal of Dox and being placed in EPO containing media (Fig. 1D).

To assess whether a longer drug-treatment regimen would lead to a similar escape phenotype, DA3/EPOR cells were treated with Dox and EPO for 72 h, and proliferation was assessed every 3 days in the presence of EPO. These cells entered a prolonged non-proliferative state and exhibited 97% SA-β-gal positivity comparable to that seen after 24 h of Dox/EPO treatment (Fig. 1E; Supplementary Fig. 1F). Under these conditions, cell proliferation was delayed until 12 days post drug treatment (Fig. 1F).

### Drug-induced senescence is independent of p53 and p16 in DA3/EPOR cells

Two core pathways that regulate DNA damage-induced senescence are the p53/p21 and p16/Rb pathways. To determine whether cells entered a p53-dependent form of senescence, a dominant negative p53 (p53DD) construct was expressed in DA3/EPOR cells (Supplementary Fig. 2A; Supplementary Fig. 12). p53DD is a C-terminal truncated isoform of p53 containing the tetramerization domain but lacking the DNA-binding domain that stabilizes and transcriptionally inactivates wild-type p53 [18]. Three independent DA3/EPOR-DD clones retained the ability to enter Dox-induced senescence similar to empty vector control cells (DA3/EPOR pcDNA) (Fig. 1G; Supplementary Fig. 2B). Previously, we reported that Nutlin-3a, a small molecule antagonist of Mdm2 stabilizes and activates p53 through a non-genotoxic process leading to the death of DA3/EPOR cells [5]. We reported that EPO attenuates the effects of nutlin-3a treatment leading to cell survival and that EPO acts in part through destabilization of the p53 protein [5]. Here, we confirm the effects of nutlin-3a on DA3/EPOR cells (Supplementary Fig. 2C). In the presence of EPO, nutlin-3a treated DA3/EPOR cells remained viable and proliferatively active, albeit at a reduced rate of proliferation compared to rapidly dividing controls (Supplementary Fig. 2C). Moreover, cells treated with nutlin-3a in the presence of EPO entered senescence on the basis of SA-β-gal staining (Supplementary Fig. 2D). These data support our finding that p53 does not play a major role in promoting senescence in DA3/EPOR cells. To assess the role of p16 in senescence, the levels of CDKN2a (p16) were measured by qRT-PCR. DA3/EPOR expressed very low levels of p16, and no increase in expression was detected when these cells entered senescence (Fig. 1H). In comparison, mouse embryonic fibroblasts (MEFs) express basal levels of p16 and these levels increase during Dox-induced senescence (Fig. 1H). Together these results indicate that DA3/EPOR cells enter senescence through a p53- and p16-independent pathway.

### Drug-recovered DA3/EPOR cells retain sensitivity to doxorubicin

Cancer cells may survive drug treatment for a number of reasons including the presence of genetic variants, non-genetic mechanisms that are associated with slow proliferation, checkpoint activation resulting in transient cell cycle arrest, and cellular senescence. The term persistent cancer cells has been used to describe cells that survive chemotherapy and exhibit a reversible slow-growing state that is not driven by any genetic variations [19]. To determine whether the drug treatment resulted in the selection of a drug-resistant clone, the drug-recovered population was re-treated with the drug for two additional rounds. Drug-recovered populations and naïve cells were treated with increasing concentrations of Dox for 48 h and cell viability was measured; no differences in cell viability were observed between the naïve parental and drug-recovered cells (Fig. 1I). To determine whether drug-recovered DA3/EPOR cells retained sensitivity to enter drug-induced senescence, we treated drug-recovered populations with Dox and EPO for 24 h and measured senescent cells 3 days after treatment using SA-β-gal staining. Drug-recovered DA3/EPOR cells all showed an increase in SA-β-gal positivity, although the drug-recovered population that went through 3 rounds of treatment showed a reduced amount of SA-β-gal cells compared to naïve cells being treated with Dox (Fig. 1J).

The cell cycle state of naïve DA3/EPOR and drug-recovered cells treated with EPO and Dox were shown by flow cytometric cell cycle analysis to be arrested in the G1 and G2/M phases of the cell cycle (day 3) and re-established a normal cell cycle profile (Supplementary Fig. 1E). Cell size increased 3 days post-treatment for both naïve and drug-recovered cells (Supplementary Fig. 1D; data not shown). Naïve and drug-recovered cells had comparable sub-G1 populations after drug treatment (Supplementary Fig. 1E). Collectively, these data show that DA3/EPOR cells resume proliferation after drug treatment. These cells retain drug sensitivity and the ability to enter a drug-induced senescent-like state, and thus, the senescent-like state facilitates transient drug tolerance.

Our results show that EPO suppresses p53-induced death and that EPO promotes entry into the senescent-like state. Moreover, continuous EPO stimulation is required for the survival of senescent DA3/EPOR cells after drug treatment (Fig. 1B–D, Supplementary Fig. 2C). We next asked if the removal of EPO during the senescent state would lead to cell death, and whether cell death resulting from EPO withdrawal was dependent on p53. The viability of senescent cells was assessed 48 h after EPO was removed from drug-induced senescent DA3/EPOR clones expressing p53DD or empty vector control. Senescent DA3/EPOR-DD clones demonstrated greater viability after the withdrawal of EPO compared with the empty vector control cells (Supplementary Fig. 2E). This suggests that p53 contributes to the death of senescent DA3/EPOR cells and is consistent with the idea that EPO-mediated repression of p53 is partially responsible for maintaining viability during drug-induced senescence.

### Drug-induced senescence in DA3/EPOR cells is reversible

**Fig. 2:**
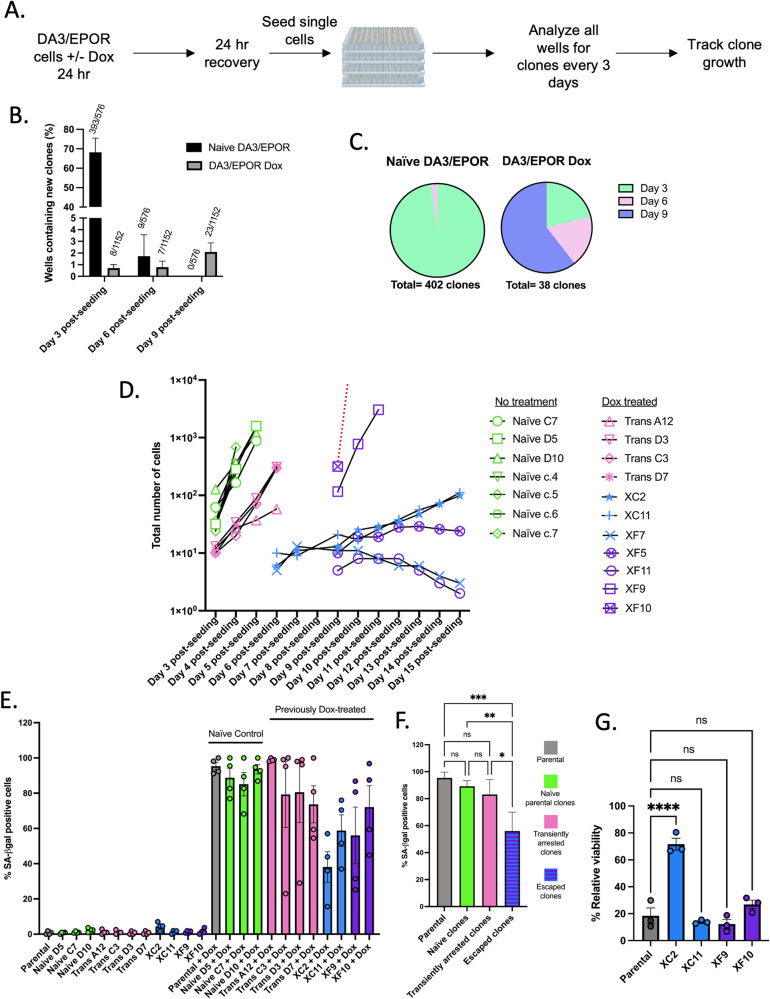
DA3/EPOR cells that escape senescence exhibit heterogeneity. **A** Experimental scheme related to **B**–**D** and Supplementary Fig. 4. Cells were treated with Dox in the presence of EPO for 24 h; naïve cells were maintained in EPO-containing media. After 24 h treatment, Dox was removed and cells were recovered for 24 h in EPO-supplemented media for 24 h. Cells were subsequently seeded into 96-well plates and monitored every 3 days for new expanding clones. Expanding clones were defined by ≥5 cells/ well. Newly formed colonies were counted every 3 days and expanding colony size was counted on indicated days to determine proliferation rates. **B** Percentage of wells containing new colonies. Mean ± SEM, *n* = 3 independent experiments. **C** Pie chart showing proportions of when new expanding clones were detected (related to **A**). **D** Rates of the proliferation of randomly selected expanding colonies. **E** SA-β-gal staining of parental DA3/EPOR, 3 naïve clone cells, 8 expanded drug-recovered clones (4 of which went through a transient arrest and 4 that escaped senescence) treated with Dox in the presence of EPO. Mean ± SEM, *n* = 4 independent experiments. F. Statistical analysis of SA-β-gal staining of the Dox-treated naïve (*n* = 3 biological samples), transiently arrested (*n* = 4 biological samples), and escaped clones (*n* = 4 biological samples) compared with Dox-treated parental cells. Related to Fig. 2E. Mean ± SEM; one-way ANOVA. **G** Relative viability of 4 escaped clones compared to parental DA3/EPOR cells treated with Dox in the absence of EPO. Mean ± SEM, *n* = 3 independent experiments; one-way ANOVA. Statistical significance is shown by *P < 0.05, **P < 0.01, ***P < 0.001, ****P < 0.0001.

To investigate whether Dox-induced senescence is reversible, we treated DA3/EPOR cells with Dox+EPO for 24 h, waited another 24 h for the cells to recover in the absence of drug, and seeded single cells into 96-well plates while monitoring wells for cell proliferation every 3 days (Fig. 2A). As noted earlier, under these same conditions, 96–99% of surviving cells enter senescence on the basis of SA-β-Gal staining. We reasoned that if Dox+EPO-treated single cells proliferate within 3 days post-seeding, then these cells likely never entered senescence but instead, may have undergone checkpoint activation and entered a transient cell cycle arrest or they may have been tolerant to the drug and never exited the cell cycle. On the other hand, if Dox + EPO-treated cells show delayed growth and only began to proliferate 3 days or more post-seeding, then these cells likely entered a senescence-like state and from which they subsequently escaped. 68.2% of wells seeded with untreated control DA3/EPOR cells contained proliferating cells within 3 days of seeding, while only 0.7% of wells seeded with Dox-treated cells contained proliferating cells within 3 days of seeding (Fig. 2B). Dox+EPO-treated cells exhibited delayed growth recovery 6 to 9 days post-seeding. Notably, of the 38 wells seeded with Dox + EPO-treated cells that showed growth, 30/38 individual cells (78.9%) showed delayed growth prior to days 6 and 9, while 9/402 untreated individual cells (2%) showed delayed growth by day 6 with the majority of the untreated cells showing growth within 3 days of seeding (Fig. 2C). These data indicate that a small fraction of cells that enter a drug-induced senescent-like state exhibit asynchronous exit.

Clonal expansion from single cells revealed extensive heterogeneity in the growth rate of escaped cells; some escaped clones expanded rapidly while others were unable to sustain proliferation (Fig. 2D) compared with naïve (never treated cells) and drug-treated cells that grew within 3 days of seeding. Proliferation rates of two escaped clones, XC2 and XC11, remained slow for >30 days in culture post-Dox treatment, and then increased to a similar rate as naïve cells. Notably, drug-treated clones that appeared within 3 days of seeding consisted of smaller colonies (fewer cells per well) compared with the naïve untreated cells supporting the view that these cells arose from transiently arrested cells that did not senesce (Fig. 2D). The proliferation rates of naïve untreated clones and transiently arrested drug-treated clones were fairly uniform, suggesting that the heterogeneity in growth observed in the escaped clones likely arose de novo as a result of drug-induced entry and exit from the senescence-like state, and did not pre-exist in the parental population.

We compared the drug sensitivity of cells derived after the expansion of individual escaped DA3/EPOR cells with cells derived from naïve and transiently arrested individual cells. Escaped clones (XC2, XC11, XF9, and XF10) showed variable drug sensitivity both in terms of drug-induced senescent-like state (Fig. 2E, F) and drug-induced cell death (Fig. 2G) while the naïve and transiently arrested clones exhibited uniform drug sensitivity similar to parental cells. To assess whether the p53 pathway remained active in the escaped clones, cells were treated with nutlin-3a in the presence or absence of EPO for 48 h. All escaped clones underwent p53-mediated death in the absence of EPO; no significant difference in the levels of death was observed between escaped clones (Supplementary Fig. 3).

To explore further the heterogenous behavior of escaped clones, three independent naïve DA3/EPOR clones (C7, D5, and D10) were subjected to Dox treatment and investigated using the single cell assay. The 3 clonal populations entered a senescence-like state similar to the parental population (Fig. 2E). Escape from a drug-induced senescence-like state occurred asynchronously for the 3 clonal populations (Supplementary Fig. 4A, B). Clonal expansion from single cells revealed extensive heterogeneity in the growth rate of escaped cells (Supplementary Fig. 4C). Like the naïve parentals, all (3/3) naïve clonal populations demonstrated the ability to persist post-drug treatment, go through transient cell cycle arrest, permanent senescence, and a reversible senescent-like state. A small proportion of senescent cells acquire the ability to escape senescence in a stochastic manner. Together these results support the idea that heterogeneity in cell behavior arises de novo after drug treatment in the escaped cells.

### Drug-induced senescence in OCIM2 human leukemia cells is reversible

OCIM2 human leukemia cell line derived from a patient with erythroleukmia was treated with the clinically relevant drug DNR for 24 h. DNR-treated OCIM2 cells entered senescence 3 days after drug treatment based on high levels of SA-β-gal positivity and increased cell size (Supplementary Fig. 5A); similar to DA3/EPOR cells, the proportion of senescent cells decreased over time. DNR-treated OCIM2 cells showed sustained proliferative arrest and delayed cell death up to 7 days post-treatment (Supplementary Fig. 5B). Drug-free recovered OCIM2 cells were able to resume proliferation after 7 days post-DNR treatment (Supplementary Fig. 5B). The ability of the mutant p53 (V274D)-expressing cell line, OCIM2, to enter senescence supports our earlier finding using the DA3/EPOR DD clones that drug-induced senescence occurs independently of p53. Drug-free recovered OCIM2 cells re-treated with DNR retained the ability to enter senescence to comparable levels as naïve OCIM2 cells (Supplementary Fig. 5C); hence, drug-free recovered OCIM2 cells are not resistant to drug-induced senescence upon further exposure to the drug. Unlike drug-free recovered DA3/EPOR cells, drug-free recovered OCIM2 cells demonstrated increased viability to DNR 3 days and 7 days post-treatment compared with naïve cells (Supplementary Fig. 5D).

To determine whether the OCIM2 cells could escape DNR-induced senescence, we performed single-cell analysis on DNR-treated OCIM2 cells and monitored individual clones for proliferation. The vast majority of naïve untreated OCIM2 cells formed colonies within 3 days post-seeding (Supplementary Fig. 6A, B). DNR-free recovered OCIM2 cells demonstrated heterogeneity; 4 of the 25 clones underwent transient or no arrest (cell expansion appearing by day 3 post-seeding), while 19/25 clones escaped DNR-induced senescence (cell expansion appearing by day 6 or day 9 post-seeding) (Supplementary Fig. 6A, B). Furthermore, escape from senescence occurred in an asynchronous manner (Supplementary Fig. 6A, B). Most naïve OCIM2 cells showed uniform rates of proliferation, whereas escaped OCIM2 cells exhibited variable rates of proliferation (Supplementary Fig. 6C) similar to what was observed in the DA3/EPOR cells. These data indicate that OCIM2 cells undergo drug-induced senescence and that a small proportion of cells are able to escape senescence asynchronously and exhibit differences in proliferation rates.

### Transcriptional reprogramming during senescence

**Fig. 3:**
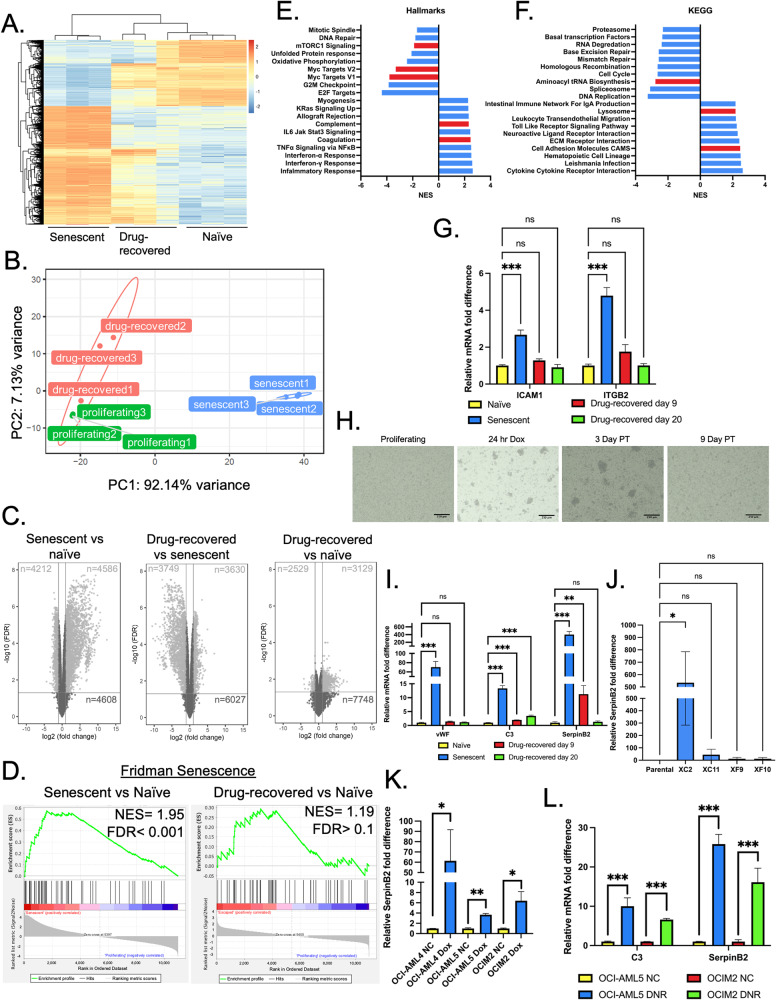
Senescent-associated changes in gene expression. **A** Heatmap of proliferating, senescent (day 3 PT), and drug-recovered (day 9 PT) DA3/EPOR cells using the top 1000 differentially expressed genes in senescent DA3/EPOR cells compared to proliferating control DA3/EPOR cells. **B** Principal component analysis (PCA) grouping proliferating control (green), day 3 senescent (blue), and day 9 drug-recovered cells (red) in biological triplicate. **C** Volcano plots showing relative gene expression of RNA-seq data. Genes with a fold change of >2 and an FDR < 0.05 were represented as light gray dots; genes with a fold change <2 and non-significant genes were displayed as dark gray dots. Total number of significantly upregulated and downregulated genes are stated in the respective quadrates. Total number of insignificant genes is stated in the bottom right quadrant. **D** GSEA plot of Fridman’s senescence in senescent DA3/EPOR cells compared to proliferating controls, and drug-recovered DA3/EPOR cells compared to proliferating controls. **E** Normalized enrichment scores (NES) of the top 10 most upregulated and downregulated Hallmarks of senescent DA3/EPOR cells compared to proliferating control cells. All Hallmarks have an FDR of >0.001. Hallmarks highlighted in red were validated. **F** NES of the top 10 most upregulated and downregulated KEGG pathways of senescent DA3/EPOR cells compared to proliferating control cells. All pathways have an FDR of >0.001. KEGG pathways highlighted in red were validated. **G** qRT-PCR analysis of ICAM1 and ITGB2 mRNA levels in proliferating, senescent, drug-recovered (9 days post-treatment), and later drug-recovered (20 days post-treatment) DA3/EPOR cells. Mean ± SEM, *n* = 3 independent experiments; one-way ANOVA. H. Representative images of proliferating, 24 h Dox treated, senescent (3 days PT), and drug-recovered (9 days PT) DA3/EPOR cells. After 24 h Dox treatment, cells were washed with 1xPBS, and cells clumped together were mechanically separated. I. qRT-PCR analysis of complement III (C3), von Willebrand factor (vWF), and SerpinB2 mRNA levels in proliferating, senescent, drug-recovered (9 days post-treatment), and later drug-recovered (20 days post-treatment) DA3/EPOR cells. Mean ± SEM, *n* = 3 independent experiments; one-way ANOVA. J. qRT-PCR analysis of SerpinB2 mRNA levels in escaped clones XC2, XC11, XF9, and XF10, that were maintained in culture for >60 days post-treatment, relative to their naïve parental controls. Mean ± SEM, *n* = 3 independent experiments; one-way ANOVA. K. qRT-PCR analysis of SerpinB2 mRNA levels in Dox-induced senescent OCI-AML4, OCI-AML5, and OCIM2 cell lines, relative to their proliferating controls. Mean ± SEM, *n* = 3 independent experiments; one-way ANOVA. L. qRT-PCR analysis of C3 and SerpinB2 mRNA levels in DNR-induced senescent OCI-AML5 and OCIM2 cell lines, relative to their proliferating controls. Mean ± SEM, *n* = 3 independent experiments; Student *t*-test. Statistical significance is shown by ns, not significantly different, *P < 0.05, **P < 0.01, ***P < 0.001.

To gain insight into the mechanism of drug-induced senescence and to evaluate differences in gene expression between drug-recovered cells and naïve proliferating cells, RNA-seq was performed on naïve, senescent, and drug-recovered DA3/EPOR cells. We collected the senescent cells on day 3 after Dox/EPO treatment; the drug-recovered cells were collected 9 days after Dox/EPO treatment. The drug-recovered population (cells that survive drug treatment and resume proliferation) consists of a mixture of cells including cells that may never have senesced as discussed above. We found that the transcriptional profiles of senescent cells were highly distinct from naïve and drug-recovered cells; drug-recovered cells resembled the naïve proliferating population (Fig. 3A–C). The volcano plot, displaying gene expression of drug-recovered proliferating cells compared with senescent cells mirrors the volcano plot of naïve proliferating cells with senescent cells, suggesting that the drug-recovered gene signature is similar to the naïve proliferating gene signature (Fig. 3C).

Gene set enrichment analysis (GSEA) showed significant enrichment for senescent genes in the Dox-induced senescent cells, and not in the drug-recovered cells (Fig. 3D). Additionally, RNA-seq demonstrated that senescent DA3/EPOR cells displayed an upregulation of multiple senescent associated secretory phenotype (SASP) genes [20], which decreased in drug-recovered cells (Supplementary Fig. 7A). p16 was not detected in any of the samples by RNA-seq (data not shown) consistent with our earlier results (Fig. 1H).

GSEA demonstrated significant enrichment for gene signatures associated with various pathways. Fig. 3E, F show the top 10 enriched and downregulated Hallmark and KEGG pathways in senescent DA3/EPOR cells compared to proliferating controls. To validate RNA-seq, several KEGG and Hallmark categories were further investigated. We included a later time point (20 days PT) to investigate the stability of the transcriptional changes of drug-recovered cells compared to proliferating cells. Consistent with the upregulation of Cellular Adhesion Molecules during senescence, qRT-PCR demonstrated that senescent DA3/EPOR cells had elevated levels of Intercellular Adhesion Molecule 1 (ICAM1) and Integrin β2 (ITGB2) compared with naïve and drug-recovered cells (Fig. 3G). DA3/EPOR cells began to form aggregates during Dox treatment and remained aggregated throughout the senescent period. Cell aggregates dispersed as cells resumed proliferation (Fig. 3H). Dox-treated DA3/EPOR cells were able to reform aggregates after being separated through pipetting (Fig. 3H). These data indicate that aggregation is associated with the senescence phenotype.

GSEA demonstrated that the coagulation and complement hallmark pathways were highly upregulated during senescence in DA3/EPOR cells. Three components of the coagulation and complement gene sets were selected for qRT-PCR analysis using RNA isolated from naïve, senescent, and drug-recovered cells. Expression of SerpinB2 (PAI2), vWF (Von Willebrand factor), and C3 (complement III) mRNA increased during senescence in DA3/EPOR cells (Fig. 3I). SerpinB2 and vWF mRNA levels returned to basal levels with prolonged time in culture. Expression of C3 remained stably elevated in drug-recovered cells on day 9 and day 20 post-treatment, indicating that although the majority of genes in drug-recovered cells return to basal levels, there are some phenotypic distinctions between drug-recovered and naïve cells (Fig. 3I).

The drug-recovered population is heterogeneous, consisting of cells proliferating after a transient cell cycle arrest, cells that have escaped senescence, and cells that remain in senescence. To determine whether the RNA-seq and qRT-PCR of the drug-recovered population reflected cells that truly escaped from drug-induced senescence, we performed qRT-PCR on four escaped clones and assessed the levels of SerpinB2, which was upregulated in drug-recovered cells. Here again, we see inter-clonal heterogeneity in the expression of SerpinB2; escaped clone XC2 displayed significantly elevated SerpinB2 relative to parental control cells and to the 3 other independent escaped clones (Fig. 3J).

To explore the possibility that SerpinB2, vWF, and C3 are potential biomarkers of therapy-induced senescence in human leukemia, 3 human AML cell lines, OCI-AML4, OCI-AML5, and OCIM2 were treated with Dox for 24 h and high levels of SA-β-gal positive cells were detected 3 days after drug treatment (Supplementary Fig. 7B). Consistent with the senescent phenotype, OCI-AML4, OCI-AML5, and OCIM2 exhibited increased cell size (Supplementary Fig. 5C). qRT-PCR analysis demonstrated that SerpinB2 mRNA levels increase significantly during Dox-induced senescence in all 3 human AML cell lines (Fig. 3K). Previous reports have suggested that SerpinB2 is a marker of senescence and may aid in the maintenance of the senescent state [21]. Dox-induced senescent OCI-AML5 cells had elevated levels of C3; expression of C3 in Dox-induced senescent OCI-AML4 and OCIM2 trended upward (Supplementary Fig. 7D). vWF was not detected in these human AML cell lines (data not shown). OCI-AML5 and OCIM2 cells were also treated with the clinically relevant drug DNR for 24 h. DNR-treated OCI-AML5 and OCIM2 cells entered senescence 3 days after drug treatment based on high levels of SA-β-gal positivity and increased cell size (Supplementary Fig. 7E, F) and expressed increased levels of SerpinB2 and C3 mRNA (Fig. 3L). We propose that SerpinB2 and C3 be considered biomarkers of chemotherapy-induced senescence in AML.

### Senescent DA3/EPOR cells mimic embryonic diapause

**Fig. 4:**
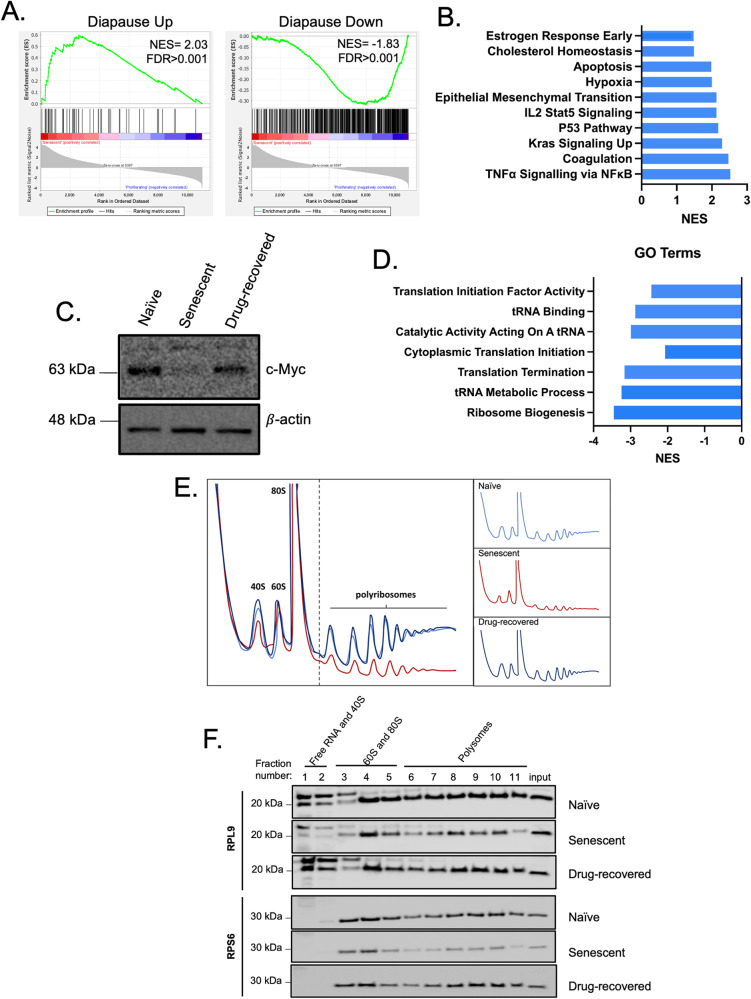
Drug-induced senescence is associated with an embryonic-diapause-like state. **A** GSEA plots of senescent DA3/EPOR cells compared to control cells using a curated diapause gene set of genes up and down-regulated in a diapause state (diapause gene set was derived from Boroviak and colleagues (2015); and is identical to the Diapause gene set used in 6; Supplementary Table 2). **B** NES of Hallmark gene sets found in a diapause state. All Hallmarks had an FDR < 0.01 (Hussein et al., 2020). **C** Representative western blot of c-Myc in proliferating, senescent, and drug-recovered DA3/EPOR cells (*n* = 2 biological replicates). **D** NES of Gene Ontology (GO) related to the translation of senescent DA3/EPOR cells compared to proliferating controls. **E** Representative traced polysome profiles of proliferating, senescent, and drug-recovered (Day 9) DA3/EPOR cells. Overlayed traced polysome profiles are on the left; individual traced polysome profiles are on the right. **F** Fractions of polysomes were taken, and protein was isolated. RPL9 and RPS6 levels were analyzed by western blot. *n* = 3 independent experiments.

Chemotherapy-treated colon carcinomas, adenocarcinoma, and acute myeloid leukemias were recently shown to enter a reversible diapause-like cell state that enabled cell survival during drug treatment [6, 12, 13]. Diapause is a reversible, evolutionary conserved, embryonic stress state, which enhances cell survival in response to environmental stress. Using GSEA and a diapause gene set [6, 22], we observed significant enrichment for diapause-associated genes in senescent DA3/EPOR cells (Fig. 4A).

Senescent DA3/EPOR cells were enriched for multiple diapause-associated hallmark pathways [23] (Fig. 4B). Inhibition of Myc and mTOR promotes a dormant state that mimics diapause [24, 25]. Both Myc and mTOR pathways were downregulated in senescent DA3/EPOR cells (Fig. 3E). Diapaused epiblasts exhibit decreased proliferation, decreased Myc expression, reduction in translation-associated genes, and reduced translation [22]. To evaluate whether senescent, non-proliferating DA3/EPOR cells were in a diapause-like state, levels of Myc expression and translation were assessed. Senescent cells demonstrated decreased c-Myc protein levels, which increased to normal levels after proliferation resumed on day 9 (Fig. 4C; Supplementary Fig. 13). GSEA demonstrated that gene ontology (GO) translation initiation and termination, as well as tRNA metabolic processes and ribosome biogenesis, were downregulated in senescent DA3/EPOR cells compared with naïve cells (Fig. 4D). Polysome analysis by sucrose gradient centrifugation, revealed that senescent DA3/EPOR cells had reduced global translation that returned to normal levels in the drug-recovered cells (Fig. 4E). Consistent with reduced translation, senescent cells displayed an altered distribution of ribosomal proteins RPL9 and RPS6 in the polysome fractions, with relatively higher levels in the free subunit and monosome fractions (Fig. 4F; Supplementary Fig. 14). Together, these data point towards an association between drug-induced senescence and the diapause state in DA3/EPOR cells.

### Lysosomal activity protects senescent leukemia cells

**Fig. 5:**
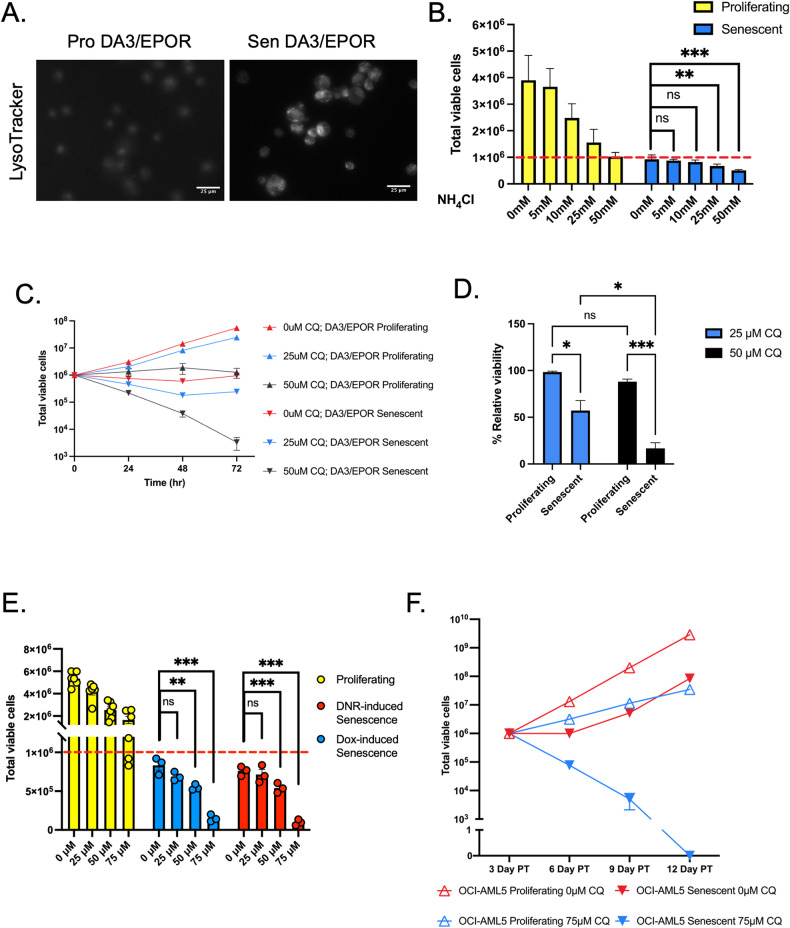
Upregulation of the lysosomal pathway promotes the survival of drug-induced senescent cells. **A** Representative immunofluorescence images of proliferating and senescent DA3/EPOR cells stained for 30 min with LysoTracker Red DND-99. **B** Viable proliferating and senescent (3 days post-dox treatment) DA3/EPOR cells were treated with increasing concentrations of NH_4_Cl or vehicle control for 24 h. The dashed red line indicates the initial number of cells seeded prior to treatment. Cell numbers that fall below the dashed red line reflect cytotoxicity. Mean ± SEM, *n* = 4 independent experiments; one-way ANOVA. **C** Senescent and proliferating DA3/EPOR cells were treated for 72 h as indicated and viable cells were counted every 24 h. Senescent cells were obtained 3 days after Dox treatment. Mean ± SEM, *n* = 3 independent experiments. **D**. Senescent and proliferating OCI-AML5 cells were treated with increasing concentrations of chloroquine (CQ) or vehicle control for 48 h. The dashed red line indicates the initial number of cells seeded prior to treatment. Cell numbers that fall below the dashed red line reflect cytotoxicity. Mean ± SEM, *n* = 6 independent experiments for proliferating cells, n = 3 for senescent cells obtained after Dox treatment, *n* = 3 for senescent cells obtained after DNR treatment; one-way ANOVA. **E** Proliferating and senescent (3 days post-DNR treatment) OCI-AML5 cells were treated with 75 μM CQ and viable cells were counted every 3 days. Mean ± SEM, *n* = 3 independent experiments. **F** Proliferating and senescent (3 days post-DNR treatment) OCI-AML5 cells were treated with 75 μM CQ and viable cells were counted every 3 days. Mean ± SEM, *n* = 3 independent experiments. Statistical significance is shown by ns, not significantly different, *P < 0.05, **P < 0.01, ***P < 0.001.

KEGG pathway analysis of the RNA-seq data indicated that the lysosome pathway was one of the highest enriched pathways during senescence and that the proteasomal pathway was downregulated (Fig. 3F). Senescent DA3/EPOR cells display an increased level of SA-β-gal activity, an indicator of lysosomal mass [26] (Fig. 1B). In addition, senescent cells stained more intensely with the LysoTracker probe than proliferating cells (Fig. 5A). As the proteasome and lysosome are integral for protein turnover and cellular homeostasis we asked whether the lysosomal pathway might be enriched to compensate for loss of the proteasome pathway.

To test whether enhanced lysosomal activity promoted survival in senescent DA3/EPOR cells, cells were treated for 24 h with NH_4_Cl, a lysosome inhibitor. Senescent DA3/EPOR cells demonstrated increased sensitivity to NH_4_Cl compared with proliferating control cells (Fig. 5B). Non-senescent cells exhibited proliferation, albeit slowed, while the senescent population demonstrated a decrease in cell number from the initial number of viable cells seeded. Next, we treated DA3/EPOR cells with chloroquine (CQ), another known lysosome inhibitor, for 72 h and observed striking senolytic activity, specifically killing senescent cells (Fig. 5C). We confirmed that the reduced number of cells treated with CQ was due to a decrease in the rate of proliferation and that CQ has specific lytic activity in senescent DA3/EPOR cells (Fig. 5D).

To examine whether CQ has similar senolytic activity on human AML cells, we treated OCI-AML5 cells with Dox or DNR to induce senescence and treated the cells with increasing concentrations of CQ. CQ exhibited senolytic activity in the senescent population (Fig. 5E). OCIM2 cells are highly sensitive to CQ and as a result, we were unable to evaluate senolytic activity in these cells (Supplementary Fig. 8). We observed that OCI-AML5 cells induced to enter senescence with DNR (0.1 μM, 24 h) are able to resume proliferation 6–9 days after drug treatment. We targeted the senescent leukemic cells with 75 μM CQ and effectively eliminated this population of cells. In contrast, proliferating OCI-AML5 cells treated with CQ had a reduced rate of proliferation, but remained viable (Fig. 5F).

### Senescence gene signature is associated with poor survival in AML and multiple other cancers

**Fig. 6:**
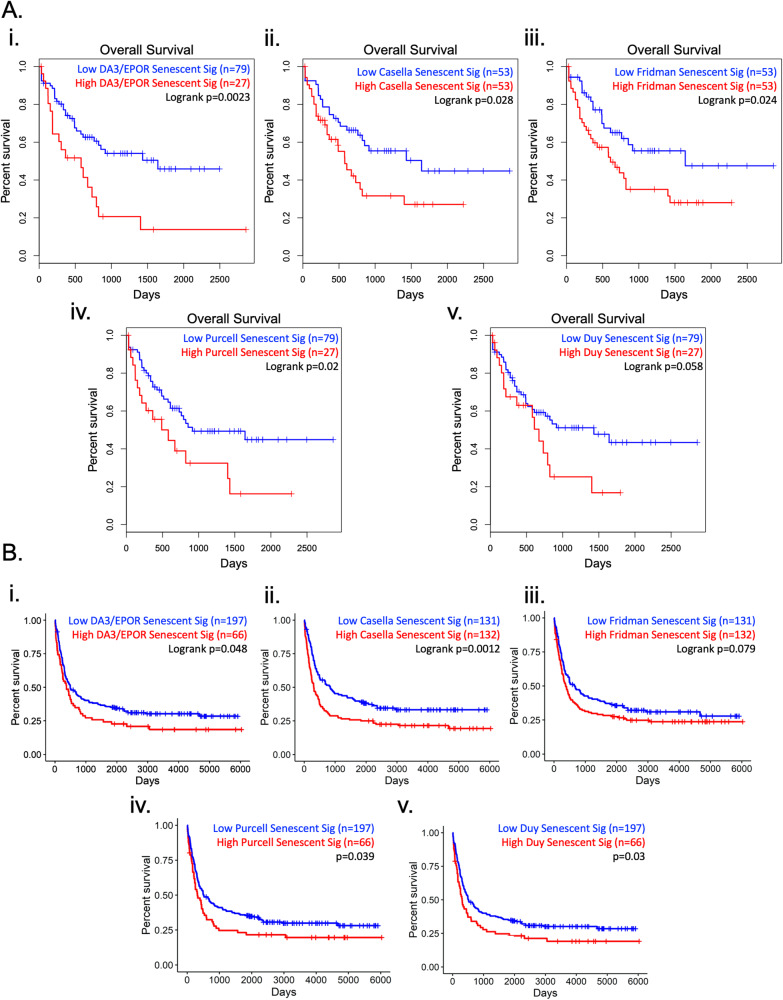
Senescence signature is associated with reduced AML patient survival. **A** Kaplan–Meier survival curves were generated on GEPIA2 using publicly available TCGA data. i. Overall survival of AML patients was segregated based on the top and bottom 3 quartiles of high and low expression of the DA3/EPOR senescent signature. The DA3/EPOR senescent signature consisted of the top 150 upregulated genes (recognized by GEPIA2) during senescence in DA3/EPOR cells compared to proliferating controls (Fig. 3D; Supplementary Table 1). ii. Overall survival of AML patients was segregated based on the median high and low expression of the Casella senescent signature [30]. Antisense and LINC RNAs were excluded from the analysis (Supplementary Table 1). iii. Overall survival of AML patients was segregated based on the median high and low expression of the Fridman senescent signature (Supplementary Table 1 [28]). iv. Overall survival of AML patients was segregated based on the top and bottom 3 quartiles of high and low expression of the Purcell senescent signature. CCDC80, CLDN1, GMPR, ITGB3, NEGR1, ODZ2, and SCN3A were excluded from the analysis, as they were upregulated in control cells (Supplementary Table 1 [29]). v. Overall survival of AML patients segregated based on top and bottom 3 quartiles of high and low expression of the Duy senescent signature (Supplementary Table 1 [6]). **B** Kaplan–Meier survival curves were generated using the BCLQ-Leucegene cohort. i. Overall survival of AML patients was segregated based on the top and bottom 3 quartiles of high and low expression of the DA3/EPOR senescent signature (Fig. 3D; Supplementary Table 1). ii. Overall survival of AML patients was segregated based on the median high and low expression of the Casella senescent signature [30]. Antisense and LINC RNAs were excluded from the analysis (Supplementary Table 1). iii. Overall survival of AML patients was segregated based on the median high and low expression of the Fridman senescent signature (Supplementary Table 1 [28]). iv. Overall survival of AML patients was segregated based on the top and bottom 3 quartiles of high and low expression of the Purcell senescent signature. CCDC80, CLDN1, GMPR, ITGB3, NEGR1, ODZ2, and SCN3A were excluded from the analysis, as they were upregulated in control cells (Supplementary Table 1 [29]). v. Overall survival of AML patients segregated based on top and bottom 3 quartiles of high and low expression of the Duy senescent signature (Supplementary Table 1 [6]).

Given the potential role of senescent cells in facilitating AML relapse [6, 7], we investigated whether a senescence gene signature may be a prognostic marker of overall survival in AML. We generated a senescence-associated gene signature curated from the most differentially expressed genes using our RNA-seq data performed on Dox-induced senescent DA3/EPOR cells. The senescent DA3/EPOR signature was created by taking the top 150 genes upregulated during Dox-induced senescence that were recognized by Gene expression profiling interactive analysis (GEPIA2) [27]. We utilized the Cancer Genome Atlas (TCGA) AML cohort and segregated the patients into two groups defined as residing within the top and bottom 3 quartiles of the senescence signature. Notably, TCGA data are derived from de novo AML patient samples prior to therapy. Kaplan–Meier analysis showed that patients with a high signature score had significantly worse overall survival than patients with a low signature score (Fig. 6Ai). Furthermore, we analyzed the same TCGA cohort of AML patients for the senescence signature derived independently in three other studies [28–30]. Notably, these senescence signatures were derived using human fibroblasts and endothelial cells. Patients were segregated into two groups, defined as having a high or low senescence signature. Kaplan-Meier analysis showed that AML patients with a high signature score had worse overall survival than patients with a low signature score (Fig. 6Aii-iv). A similar correlation with AML survival was found using a previously published senescent AML-associated gene signature (Fig. 6Av; 6).

To further investigate whether the senescence signatures were associated with reduced overall survival in AML, we employed the BCLQ-Leucegene cohort of 263 sequenced samples collected from newly diagnosed patients with de novo AML treated with intensive chemotherapy [31]. We interrogated the BCLQ-Leucegene cohort with each respective senescence-associated gene signature using the same cut-offs that were applied to the TCGA AML cohort. High expression of our DA3/EPOR senescence signature had significantly worse overall survival in the BCLQ-Leucegene cohort compared with the low signature group (Fig. 6Bi). Interestingly, other senescence signatures derived from non-AML cells also showed an association with worse overall survival in the BCLQ-Leucegene cohort (Fig. 6Bii-v). In addition, we inquired whether the senescence signatures are prognostic in the BeatAML cohort but were unable to show an association in either direction within this cohort using all 5 senescent gene signatures (Supplementary Fig. 9A–E).

We extended our analysis to various cancers of the TCGA and found that a high senescence signature [28] corresponds to poor overall survival in some but not all cancers (Supplementary Fig. 10A, B).

## Discussion

In this study, we used an experimental cell model to show that chemotherapy induces reversible senescence in myeloid leukemia, allowing some cells to resist drug treatment and resume proliferation when the drug is removed. How escaped cells differ from senescent and naïve cells is largely unknown. We show that the naïve parental DA3/EPOR population and naïve clones derived from single cells can enter drug-induced senescence. A small fraction of cells that enter drug-induced senescence exhibit asynchronous exit from senescence. Clonal expansion from single cells revealed extensive heterogeneity in the growth rate of escaped cells. Moreover, escaped clones exhibit different sensitives to a second round of drug treatment. We also find inter-clonal variability in the expression of a senescence-associated gene, SerpinB2.

Based on the single-cell assays, autonomous escape from senescence appears to be a rare event occurring in an asynchronous manner several days post-treatment; it remains possible that cells may be to escape senescence after longer periods of time that were assessed. In contrast, the pooled drug-recovered population of DA3/EPOR cells resumes proliferation within 4 days post-treatment. The drug-recovered population that resumes proliferation is likely to be composed of cells that were never arrested, cells that went through a transient arrest, and cells that recovered from senescence. Our single-cell assay demonstrates that cells can escape senescence in a spontaneous autonomous manner. However, we cannot overlook the possibility that the pooled drug-treated population can act in a paracrine-like manner to facilitate the escape of senescent cells or influence the rate of escape.

Three highly expressed genes in senescent cells include vWF, C3, and SerpinB2, components of the coagulation and complement hallmark gene sets. Complement effectors directly enhance coagulation and complements inhibit anti-coagulant factors. C3, a gene playing a central role in the complement cascade and a part of the coagulation gene set, was the only validated gene to remain stably upregulated in the drug-recovered population, demonstrating that the drug-recovered population retains some senescence-associated phenotypes and are distinct from naïve proliferating controls. The interactions of complement with other inflammatory mediators can increase the thrombogenicity of blood [32]. Using C3−/− and C5−/− mice models, Subramaniam and colleagues (2017) reported that complement proteins participate in venous thrombosis after ligation of the inferior vena cava [33]. The activation of SASP in senescent cells may also contribute to thrombosis [34]. Thrombosis occurs in up to 10% of patients with newly diagnosed AML with most thrombotic events occurring before the start of the second course of chemotherapy [35]. In addition, 28% of patients with multiple myeloma undergoing chemotherapy experience deep vein thrombosis [36]. The possible contribution of drug-induced senescent cells to thrombosis in patients undergoing chemotherapy is intriguing and warrants further investigation.

Consistent with our previous study, we find that EPO mediates the survival of DA3/EPOR cells and allows cells to persist through drug treatment [5]. Senescent DA3/EPOR cells enter dormancy with reduced translation and expression of c-MYC that resembles a diapause state. Transcriptional profiles of senescent DA3/EPOR cells show parallels with diapause epiblasts. A diapause-like state has been recently shown to promote a reversible state of drug persistence in colon cancer and AML [6, 13]. The molecular mechanisms that enable cancer cells to persist through drug treatment are largely unknown. In our study, the escaped clones retained sensitivity to drug treatment and retained the ability to re-enter senescence, indicating that resistance to drug treatment was a transient response. We propose a non-genetic mechanism in which cells gain transient resistance. We show that DA3/EPOR cells enter a drug-induced senescent state that mimics diapause. Survival of cells post-therapy in the senescent, diapause-like state partially depends on continued EPO treatment. Traditionally, senescence has been viewed as a tumor-suppressive barrier that halts the progression of tumor formation. We propose that tumor cells can exploit the senescent phenotype to persist throughout the duration of chemotherapy. Persistent cancer cells can utilize multiple, non-mutually exclusive, evolutionarily conserved stress responses to survive therapy [19].

The mechanism underlying the deleterious effects observed in cancer patients receiving EPO-stimulating agents to treat anemia is not well understood. EPO, acting through the canonical EPO-R or through ephrin-type B receptor 4 (EphB4), an alternative EPO-R, may account for the increased tumor growth and progression through downstream activation of the Src-STAT3 pathway seen in cancer patients treated with ESAs [37]. EPO-R and EphB4 are expressed in a wide range of tissues, hematopoietic and nonhematopoietic cancers at the RNA and protein level [38–40]. A remarkable degree of heterogeneity in EPOR expression is found in AML (Supplementary Fig. 10). Our findings suggest that ESAs may contribute to tumor progression and decreased patient survival by promoting and sustaining cellular senescence in response to chemotherapy.

We are uncertain why the senescence gene signatures were not prognostic in the BeatAML cohort in contrast with the TCGA and BCLQ-Leucegene cohorts. The BeatAML cohort is different from the TCGA and the BCLQ-Leucegene cohorts, as the TCGA and BCLQ-Leucegene studies consisted of samples taken from de novo AML at the time of diagnosis, whereas the BeatAML study consisted of samples taken from patients with myeloproliferative neoplasms, different forms of AML including AML that transformed from a background of myelodysplastic syndrome and AML at various stages of disease (diagnosis, residual, relapse, post-therapy) [41, 42]. An additional factor that likely influenced the difference in the prognostic ability of the senescent gene signatures was that patients within the cohorts had different treatment regimens.

AML cells emerging from therapy-induced senescence may contribute to patient relapse, seen in 40–50% of AML patients [43]. Clinical studies report that chemotherapy promotes senescence in AML [6] and in other cancers [44]. Heterogeneity within the drug-free recovered population may contribute to increased aggression in relapsed patients [45]. Although senescent cells are resistant to Dox-induced apoptosis, senolytic drugs can be used to selectively eliminate senescent cells [46]. Here we show that the lysosome pathway is upregulated during therapy-induced senescence and this promotes the survival of DA3/EPOR cells; lysosome inhibition and cytotoxic drug treatment display synthetic lethality in certain mouse and human leukemic cell lines. CQ, an FDA-approved anti-malaria drug, can be used to inhibit lysosome function and may act as a senolytic for therapy-induced senescence cells in AML patients undergoing chemotherapy. Eliminating therapy-induced senescent cells may decrease the detrimental effects of SASP and the frequency of AML relapse.

## Methods

### Cell Culture

DA3/EPOR cells and derivative clones [5, 17] were maintained at 37 °C with 5% CO_2_ in RPMI 1640 media supplemented with 10% fetal bovine serum (FBS) and 1 U/ml recombinant human erythropoietin (EPO). OCI-AML4, OCI-AML5, and OCIM2 cells were obtained from Dr. Mark Minden (University Health Network, Toronto); these cells were maintained at 37 °C with 5% CO_2_ in α-MEM with 10% FBS. Drug treatment, proliferation assays, senescence staining, and flow cytometry are described in detail in the Supplementary Methods.

### Single-cell cloning

DA3/EPOR cells were treated with Dox or vehicle control (ddH_2_O) for 24 h and allowed to recover for 24 h in drug-free media. A serial dilution was performed and single cells were seeded into wells of a 96-well plate. All wells were assessed for colony formation every 3 days. Colonies were defined as ≥5 cells in a well. Cells in wells containing colonies were manually counted directly under the microscope or on imageJ.

### RNA expression

RNA expression was measured by qRT-PCR using the primers listed in the Supplementary Methods.

### RNA-seq analysis

RNA-sequencing (RNA-seq) libraries were constructed from RNA isolated from DA3/EPOR cells and subjected to Illumina sequencing and analysis as described in the Supplementary Methods.

### Protein expression

Protein expression was measured by immunoblotting using the antibodies listed in the Supplementary methods.

### Polysome analysis

Polysome profiling was performed by fractionation as described (https://bio-protocol.org/e833). The procedure is described in detail in the Supplementary Methods.

### SA-β-gal staining

Senescence-associated β-gal (SA-β-gal) staining was performed as previously reported [47]. The procedure is described in detail in the Supplementary Methods.

### Survival analysis

Kaplan–Meier survival curves using the TCGA data were performed using Gene Expression Profiling Interactive Analysis 2 (GEPIA2). The processed BeatAML and BCLQ-Leucegene sequencing data was converted from RPKM to TPM. The log_2_(TPM + 1) was considered for the analysis, consistent with the analysis performed in GEPIA2. Leucegene RNA-sequencing data for the following samples: GSE49642, GSE52656, GSE62190, GSE66917, and GSE67039, was integrated and assessed for potential batching using principal component analysis. Multiple test corrections to control the false discovery rate using the Benjamin and Hochberg method. Kaplan–Meir survival curves were plotted for groups of patients for the binary signatures based on quartile or median cut-offs. A log-rank test was performed to determine the p-value.

### Statistical analysis

GraphPad Prism 9 was used to determine statistical significance. Two-way Student’s *t*-test (to compare means of two samples different samples), by one-way ANOVA (to compare means of more than two different samples), or by two-way ANOVA (to compare the means of samples influenced by two independent variables) was performed to determine *P* values. One-way ANOVA was followed by either Dunnett’s method (to compare multiple means to a single control mean), or Tukey’s method (to compare all means to each other). Data graphed on GEPIA2 was statistically analyzed using default GEPIA2 settings and tests.

### Research reproducibility

Computer code is available on GitHub: bhklab/leukemia-heterogeneity-analysis: Heterogeneity in leukemia cells that escape drug-induced senescence-like state (github.com)

The Leucegene data can be accessed via: https://www.ncbi.nlm.nih.gov/geo/query/acc.cgi?acc=GSE67040.

## Supplementary information

Supplementary Figure captions

Supplemental Figure 1

Supplemental Figure 2

Supplemental Figure 3

Supplemental Figure 4

Supplemental Figure 5

Supplemental Figure 6

Supplementary Figure 7

Supplementary Figure 8

Supplementary Figure 9

Supplementary Figure 10

Supplementary Figure 11

Supplementary Table 1

Supplementary Table 2

Original data files

Supplementary information

Check list

## Data Availability

RNA-seq data can be accessed at: https://www.ncbi.nlm.nih.gov/geo/query/acc.cgi?acc=GSE186200 with the token: ezabmigiplqxded.
