# Supplementary material for: Heterogeneity in leukemia cells that escape drug-induced senescence-like state
Source: Cell Death Dis. 2023 Aug 5;14(8):503. doi: 10.1038/s41419-023-06015-4 (PMC10404232; doi:10.1038/s41419-023-06015-4)
Supplement: Supplementary file 1 — Supplementary Figure captions [file 41419_2023_6015_MOESM1_ESM.docx]

**Supplementary Figures**

**Supplementary Figure 1**

1. Schematic of DA3/EPOR cells being treated with Dox and EPO for 24 hr. Related to Figure 1A, C.
2. Representative images of SA-β-gal stain of DA3/EPOR cells 3 and 9 days post-treatment. DA3/EPOR cells treated with or without EPO in the presence of absence of Dox for 24 hours. Related to Figure 1A. Scale bars are shown in the bottom right of each panel.
3. Quantification of SA-βgal stain of DA3/EPOR cell 3, 6 and 9 days post-24 hour Dox treatment (senescent), and negative control (proliferating) cells maintained in media containing EPO. Error bars represent SEM, n=3 biological replicates; a minimum of 100 cells were counted for each biological replicate.
4. FACs analysis of DA3/EPOR cells and drug-recovered DA3/EPOR cells treated with Dox for 24 hr in complete media. Cells were harvested immediately after 24 hr treatment or allowed to recover for 3 days post-treatment in complete media. n=2 independent experiments. FACs analysis demonstrating granularity and cell size.
5. Cells treated as in (D) were stained with propidium iodide to measure DNA content and cell cycle profiles.
6. Schematic of DA3/EPOR cells being treated with Dox and EPO for 72 hr. Related to Figure 1D-E.

**Supplementary Figure 2**

1. Western blot of DA3/EPOR DD expressing clones. FL393 p53 polyclonal antibody shows stabilization of full length wt-p53 and high expression of DD fragments in C16, C18 and C19 clones. β-actin was used as a loading control. n= 2 independent experiments.
2. Representative images of SA-β-gal stain of DA3/EPOR p53 dominant-negative expressing clones C16, C18 and C19, and pcDNA empty vector control, 0, 3 and 9 days post-24 hr Dox treatment. Related to Figure 1F. Scale bars are shown in the bottom right of each panel.
3. Proliferation of DA3/EPOR treated with DMSO or 10 μM nutlin-3a for 72 hr in the presence or absence of EPO. Cells were counted every 3 days.
4. SA-β-gal staining of DA3/EPOR cells treated with DMSO or with10 μM Nultin-3a for 72 hr in the presence of EPO. Mean ± SEM, n = 3 independent experiments; two-way student t-test. A minimum of 100 cells were counted for each independent experiment.
5. Percentage of viable senescent and proliferating DA3/EPOR DD-expressing clones and pcDNA control cells after having EPO withdrawn for 48 hr relative to cells maintained in EPO containing media. Mean ± SEM, n = 4 independent experiments for pcDNA, C16 and C19, n=3 for C18; two-way ANOVA.

**Supplementary Figure 3**

Viability of escaped clones (XC2, XC11, XF9. And XF10) and parental DA3/EPOR cells treated with nultin-3a relative to DMSO treatment. Mean ± SEM, n = 3 independent experiments; two-way ANOVA.

**Supplementary Figure 4**

1. Percentage of wells containing new colonies of naïve clones. Mean ± SEM, n = 3 independent experiment.
2. Pie chart showing proportions of when new expanding clones were detected (related to supplementary figure 4A).
3. Rates of proliferation of randomly selected expanding colonies. Related to supplementary figure 4A. n=3 independent experiments.

**Supplementary Figure 5**

1. Representative images and quantification of SA-βgal stain of OCIM2 cell 3, and 12 days post-24 hour 0.25μM DNR treatment and negative control (naïve) cells. Mean ± SEM, n = 3 independent experiments; two-way ANOVA. A minimum of 100 cells were counted for each independent experiment.
2. Proliferation curves of OCIM2 cells treated with 0.25μM DNR for 24 hr or vehicle control (ddH_2_O). After 24 hr treatment, cells were maintained in drug-free media. Cells were counted every 2 days. Mean ± SEM, n = 3 independent experiments.
3. Quantification of SA-βgal stain of naïve and drug-recovered OCIM2 cell 3 days post-24 hr DNR treatment. A minimum of 100 cells were counted for each sample. Mean ± SEM, n = 4 independent experiments; two-way ANOVA. A minimum of 100 cells were counted for each independent experiment.
4. Naïve and drug-recovered OCIM2 cells were treated with 0.25μM DNR for 24 hr or vehicle control (NC). Viability was measured after 24 hr treatment, 3 days post-treatment and 7 days post-treatment. Mean ± SEM, n = 3 independent experiments; two-way ANOVA. A minimum of 100 cells were counted for each independent experiment.

Statistical significance is shown by ns, not significant, *P<0.05, ****P<0.0001.

**Supplementary Figure 6**

1. Percentage of wells containing new colonies of naïve clones. Mean ± SEM, n = 3 independent experiment.
2. Pie chart showing proportions of when new expanding clones were detected (related to supplementary figure 6A).
3. Rates of proliferation of randomly selected expanding colonies. Related to supplementary figure 6A. n=3 independent experiments.

**Supplementary Figure 7**

1. Heat map displaying RNA-seq regulation of Coppé’s senescence-associated secretory gene set (20) in proliferating, senescent and drug-recovered DA3/EPOR cells.
2. Representative images and quantification of SA-βgal stain of human AML4, AML5 and OCIM2 cells 72 hours post-24 hour Dox treatment (senescent), and negative control (proliferating) cells. Student t-test was performed to determine statistical significance (n= 3 biological replicates). Scale bar shown in bottom right.
3. Diameter of NC and Dox treated OCI-AML4, OCI-AML5 and OCIM2 cells measured from 3 independent experiments (OCI-AML4 Proliferating n= 162, OCI-AML4 Senescent n=167; OCI-AML5 Proliferating n=160, OCI-AML5 Senescent n=161; OCIM2 Proliferating n=156, OCIM2 Senescent n=179). A minimum of 50 cells were measured for each independent experiment. The red line indicates the median diameter. Student t-test was performed using the means of each independent replicate; n= 3 biological replicates.
4. Relative mRNA expression of C3 by qRT-PCR of Proliferating and Dox-treated OCI-AML4 and OCIM2 cells. Mean ± SEM, n = 3 independent experiments; Two-way student t-test.
5. Quantification of SA-βgal stain of human AML5 and OCIM2 cells 72 hours post-24 hour DNR treatment (senescent), and negative control (proliferating) cells. Mean ± SEM, n = 3 independent experiments; Two-way student t-test. A minimum of 100 cells were counted for each independent experiment.
6. Diameter of NC and DNR treated OCI-AML5 and OCIM2 cells measured from 3 biological experiments (OCI-AML5 Proliferating n= 167, OCI-AML5 Senescent n=158; OCIM2 Proliferating n= 167, OCIM2 Senescent n= 147). A minimum of 100 cells were measured for each independent experiment. The red line indicates the median diameter. Student t-test was performed using the means of each independent replicate; n= 3 biological replicates.

Statistical significance is shown by ns, not significant, *P<0.05, **P<0.01, ***P<0.001.

**Supplementary Figure 8**

Viable proliferating and senescent (3 days post-Dox or DNR treatment) OCIM2 cells treated with increasing concentrations of chloroquine (CQ) or vehicle control for 48hr. The dashed red line indicated the initial number of cells seeded prior to treatment. Mean ± SEM, n = 6 independent experiments for proliferating cells, n = 3 for senescent cells obtained after Dox treatment, n = 3 for senescent cells obtained after DNR treatment; one-way ANOVA.

**Supplementary Figure 9**

A-E) Kaplan-Meier survival curves were generated using the BeatAML cohort.

1. Overall survival of AML patients segregated based on top and bottom 3 quartiles of high and low expression of the DA3/EPOR senescent signature (Figure 3D; Supplementary Table 1).
2. Overall survival of AML patients segregated based on median high and low expression of the Casella senescent signature (30). Antisense and LINC RNAs were excluded from the analysis (Supplementary Table 1).
3. Overall survival of AML patients segregated based on median high and low expression of the Fridman senescent signature (Supplementary Table 1; 28).
4. Overall survival of AML patients segregated based on top and bottom 3 quartiles of high and low expression of the Purcell senescent signature. CCDC80, CLDN1, GMPR, ITGB3, NEGR1, ODZ2, and SCN3A were excluded from the analysis, as they were upregulated in control cells (Supplementary Table 1; 29).
5. Overall survival of AML patients segregated based on on top and bottom 3 quartiles high and low expression of the Duy senescent signature (Supplementary Table 1; 6).

**Supplementary Figure 10**

1. Overall survival of Adrenocortical carcinoma (ACC), Bladder Urothelial Carcinoma (BLCA), Brain Lower Grade Glioma (LGG), Cervical squamous cell carcinoma and endocervical adenocarcinoma (CESC), Liver hepatocellular carcinoma (LIHC), Lung squamous cell carcinoma (LUSC), Kidney renal papillary cell carcinoma (KIRP), Mesothelioma (MESO), and Uveal Melanoma (UVM) patients segregated based on the median high and low expression Fridman’s senescent signature (Supplementary Table 1).
2. Disease-free survival of ACC, BLCA, and LGG patients segregated based on the median high and low expression Fridman’s senescent signature (Supplementary Table 1).

**Supplementary Figure 11**

RNA expression of EPOR in AML derived tumour (T) samples (N=171) compared to normal (N) bone marrow samples (N=70). GEPIA2 was used to generate expression data using tumour data from TCGA and normal tissue data from the Genotype-Tissue Expression (GTEx) project.

**Supplementary Figure 12**

Unedited westernblot images of the empty vector pcDNA DA3/EPOR cells, and DD expression clones (C16, C18, and C19) from left to right from the ladder. Images used as representative westernblot of p53 and 𝛽actin corresponding to Supplementary Figure 2A. Red arrows point to full-length p53 and DD fragments. Yellow arrow points to 𝛽actin.

**Supplementary Figure 13**

Unedited westernblot images of naive proliferating, senescent and drug recovered DA3/EPOR cells, in order from right to left of the ladder. Highlighted in red are the images used as representative c-myc and 𝛽-actin corresponding to Figure 4C. Highlighted in yellow is a biological replicate ran on the same gel, demonstrating similar results.

**Supplementary Figure 14**

Raw unedited western blots of polysome profile fraction of naive, senescent, and drug-recovered DA3/EPOR cells corresponding to Figure 4F.
