# Supplementary figures and images for: Heterogeneity in leukemia cells that escape drug-induced senescence-like state
Source: Cell Death Dis. 2023 Aug 5;14(8):503. doi: 10.1038/s41419-023-06015-4 (PMC10404232; doi:10.1038/s41419-023-06015-4)

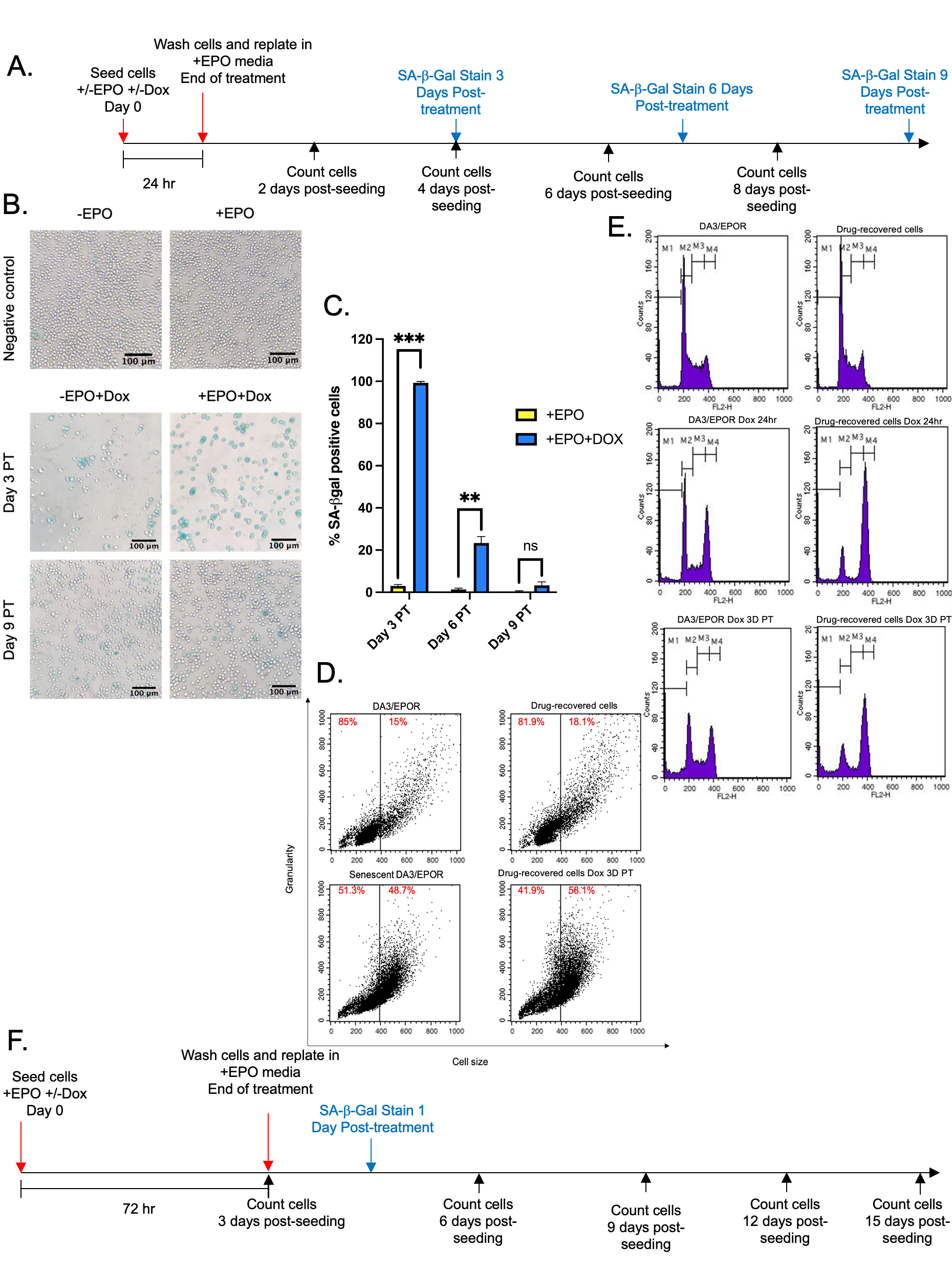

Supplement: Supplementary file 2 — Supplemental Figure 1 [file 41419_2023_6015_MOESM2_ESM.tif]

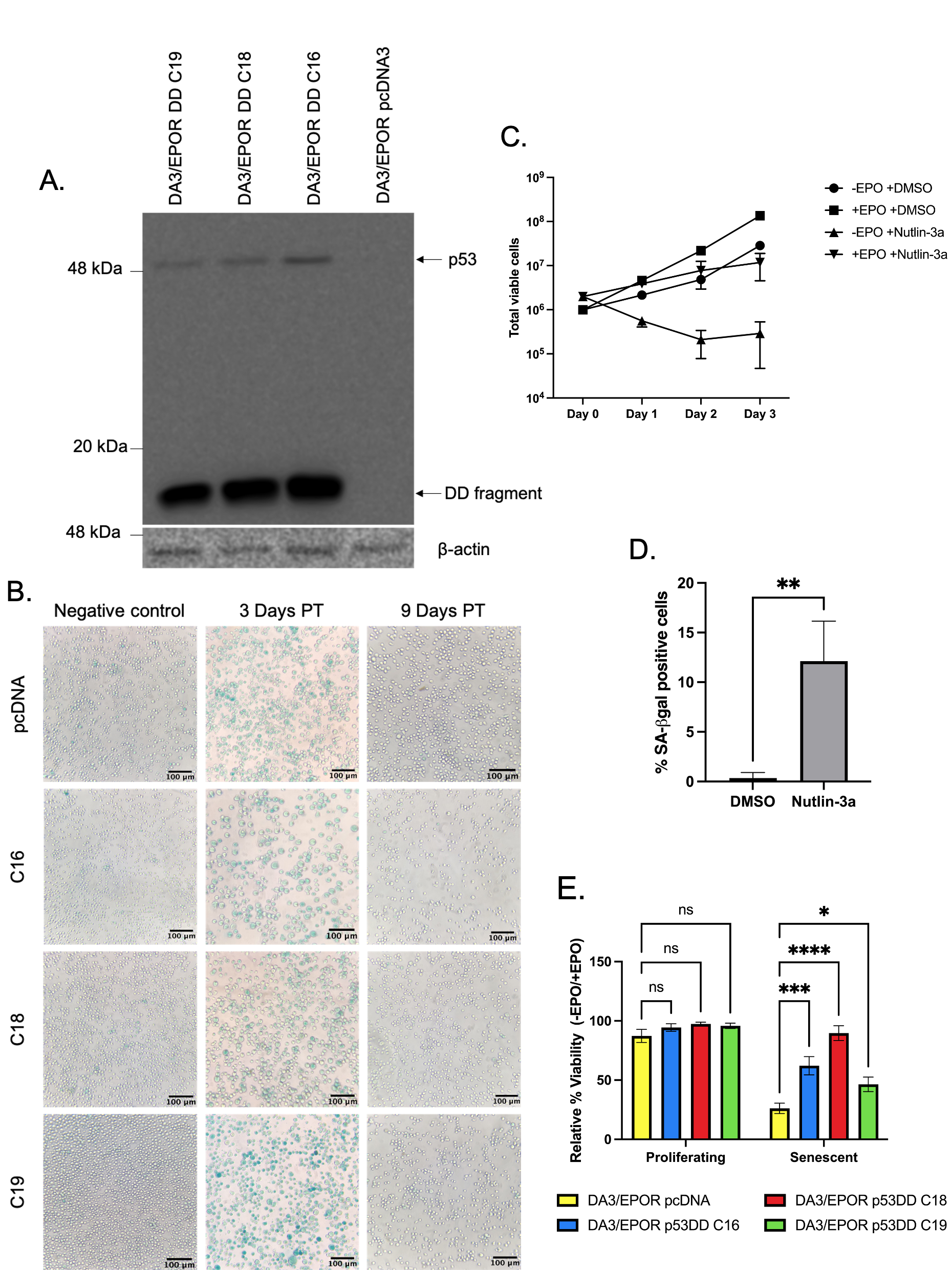

Supplement: Supplementary file 3 — Supplemental Figure 2 [file 41419_2023_6015_MOESM3_ESM.tif]

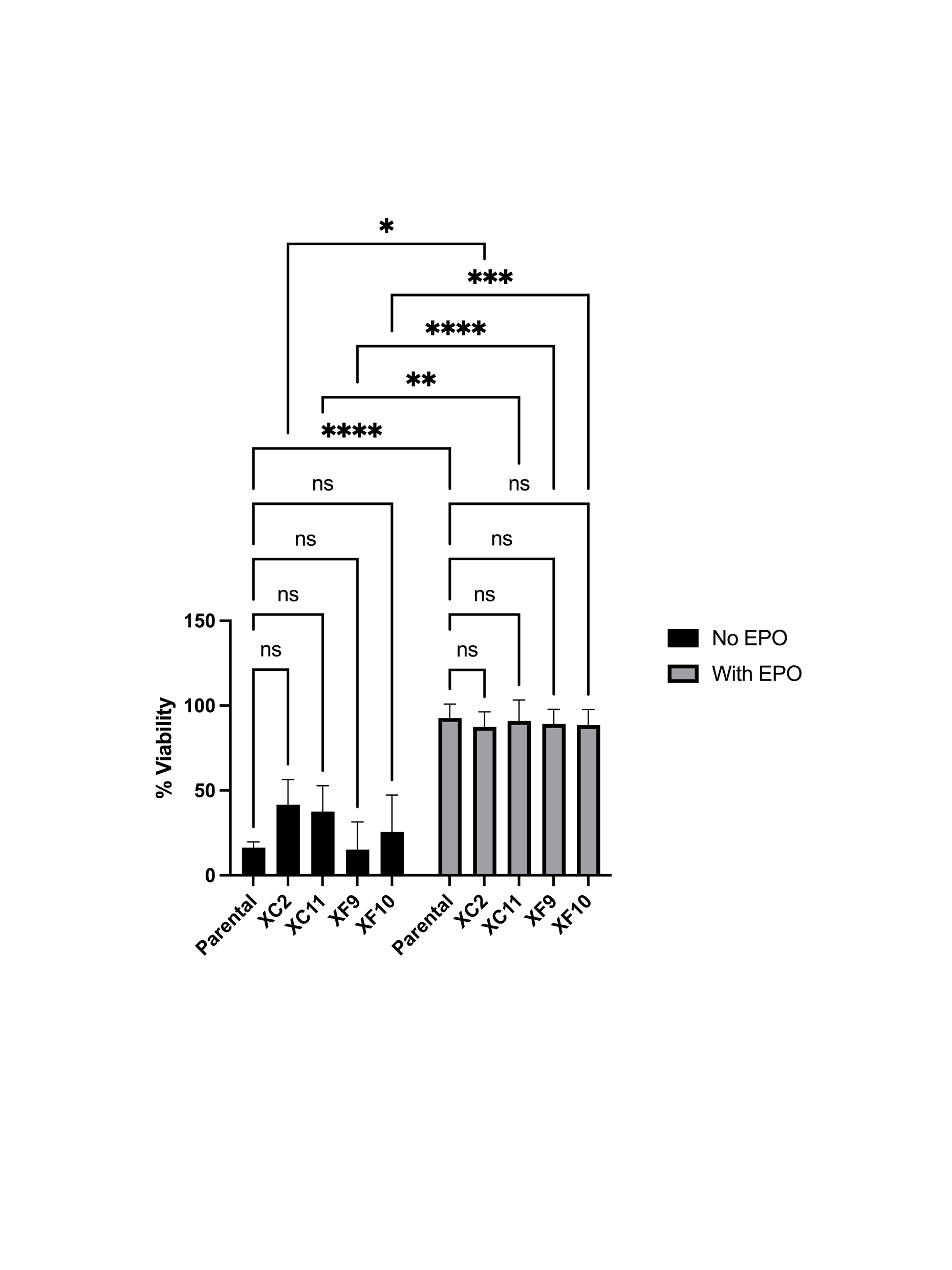

Supplement: Supplementary file 4 — Supplemental Figure 3 [file 41419_2023_6015_MOESM4_ESM.tif]

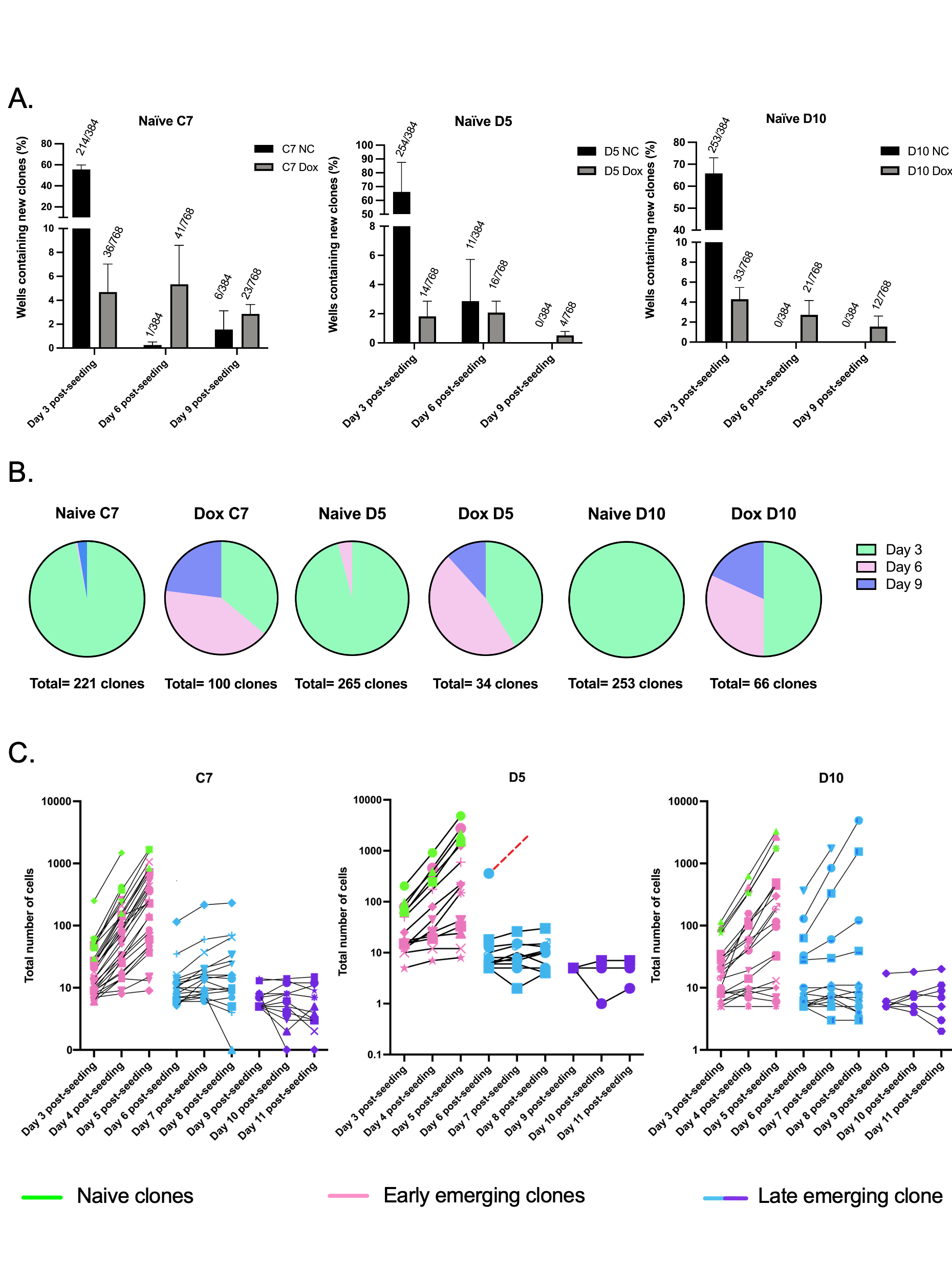

Supplement: Supplementary file 5 — Supplemental Figure 4 [file 41419_2023_6015_MOESM5_ESM.tif]

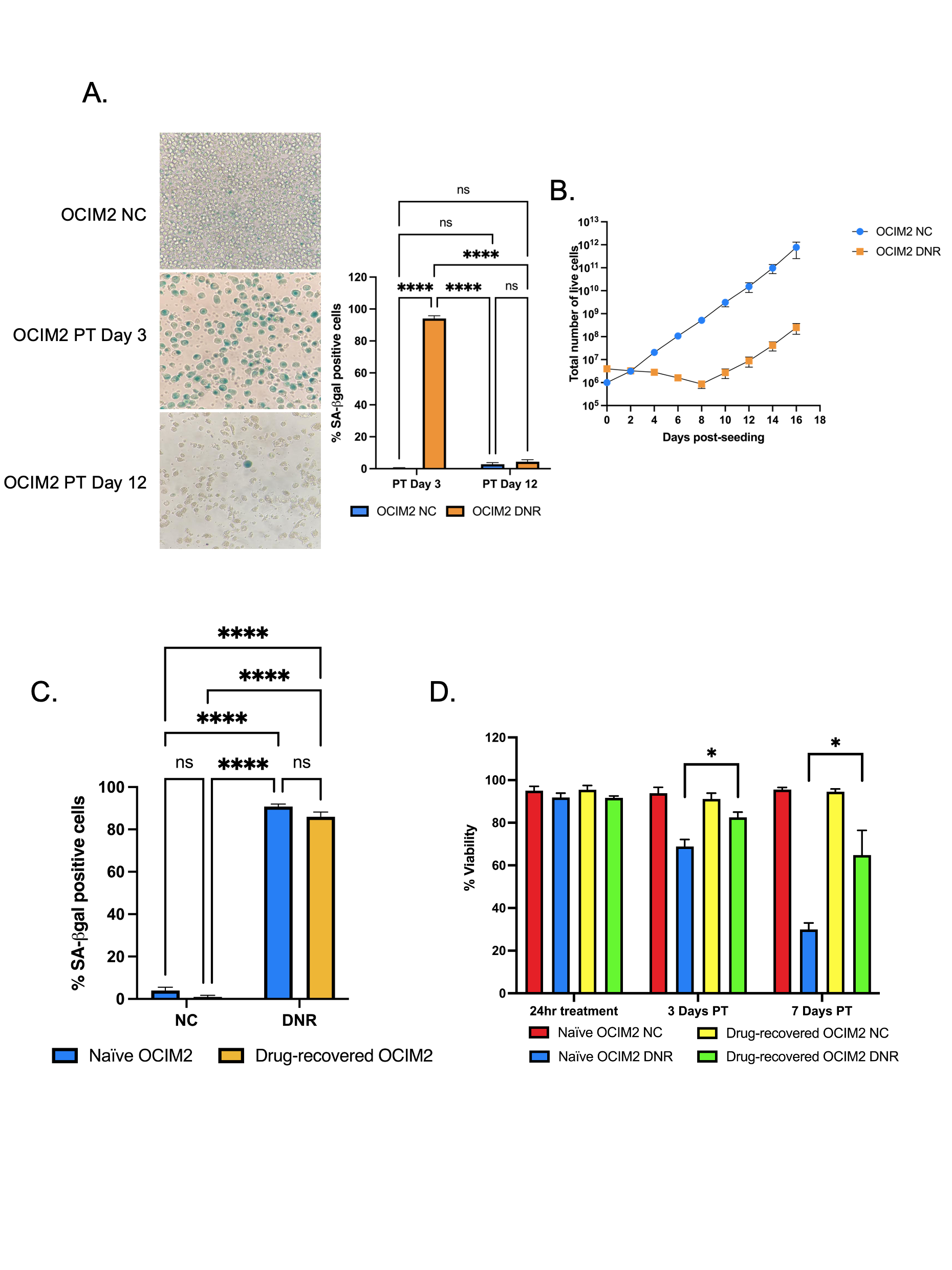

Supplement: Supplementary file 6 — Supplemental Figure 5 [file 41419_2023_6015_MOESM6_ESM.tif]

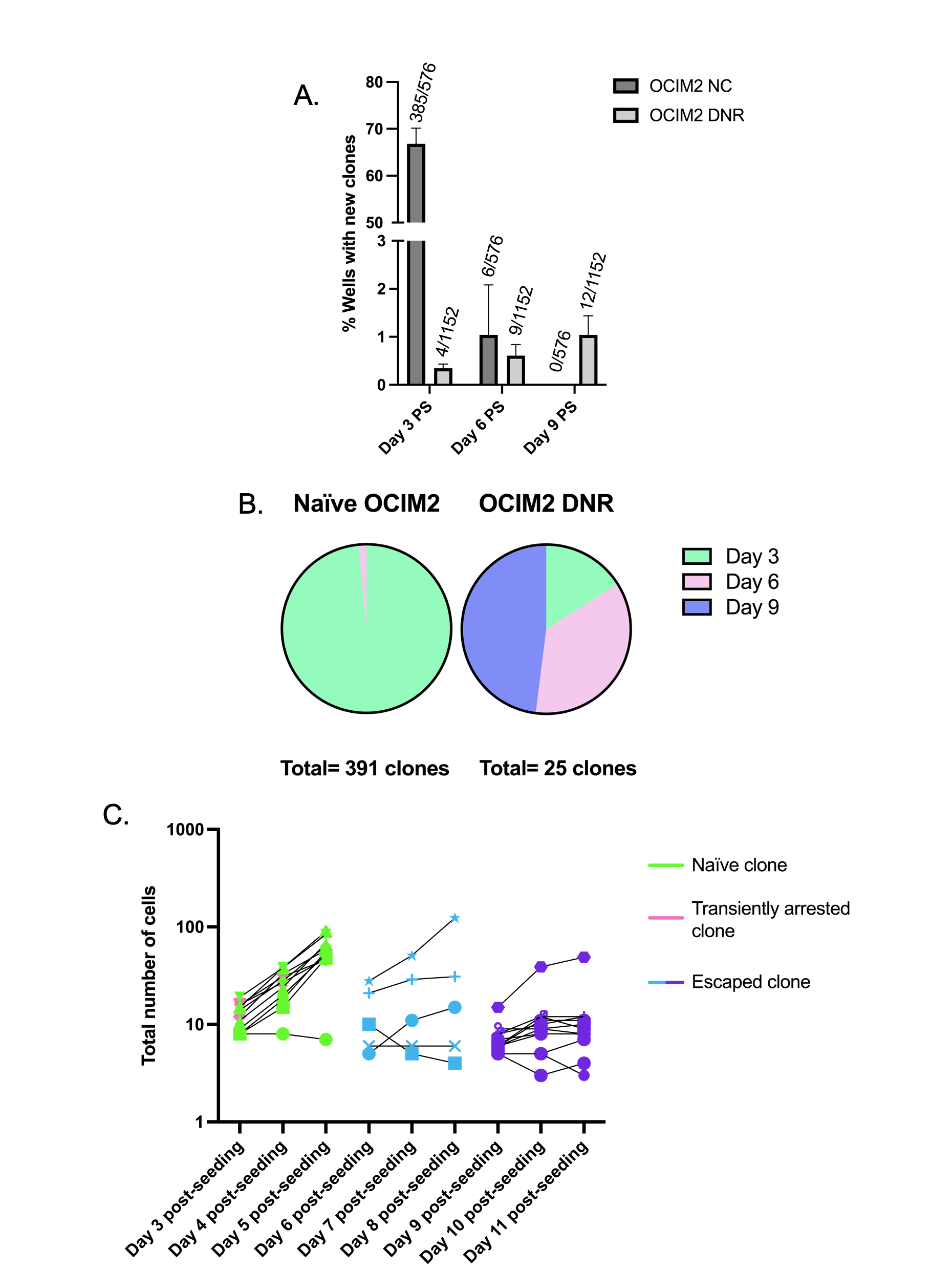

Supplement: Supplementary file 7 — Supplemental Figure 6 [file 41419_2023_6015_MOESM7_ESM.tif]

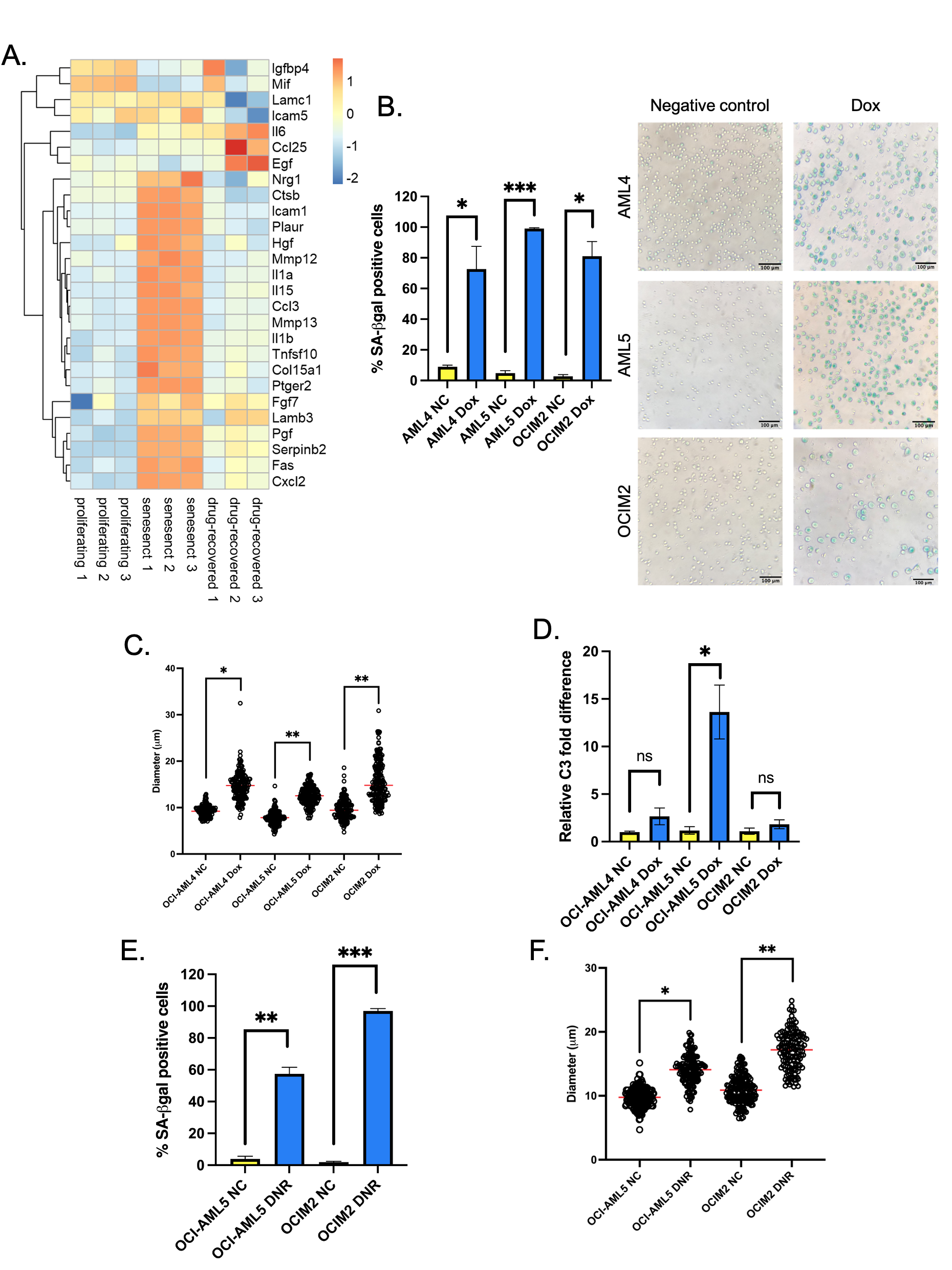

Supplement: Supplementary file 8 — Supplementary Figure 7 [file 41419_2023_6015_MOESM8_ESM.tif]

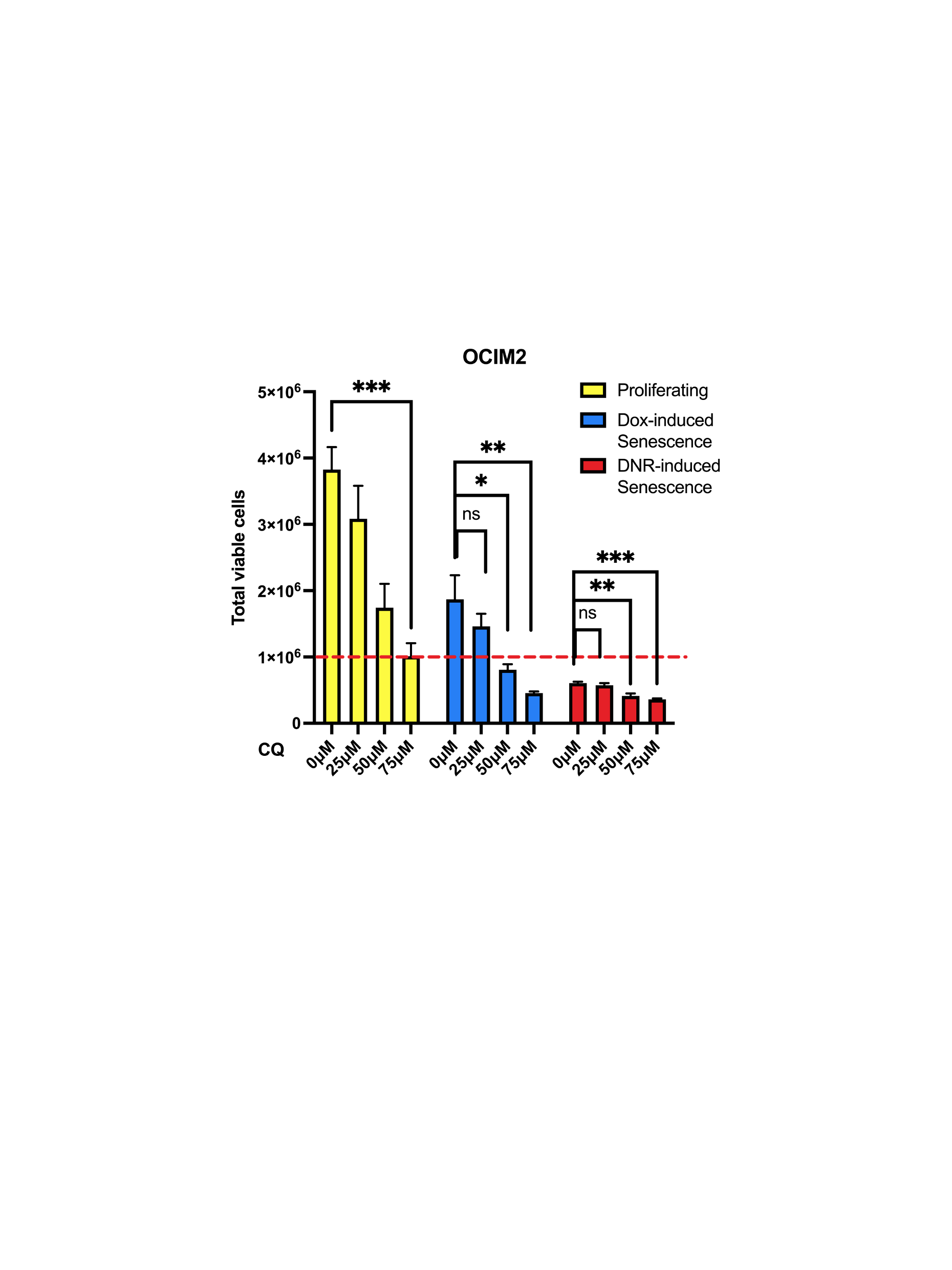

Supplement: Supplementary file 9 — Supplementary Figure 8 [file 41419_2023_6015_MOESM9_ESM.tif]

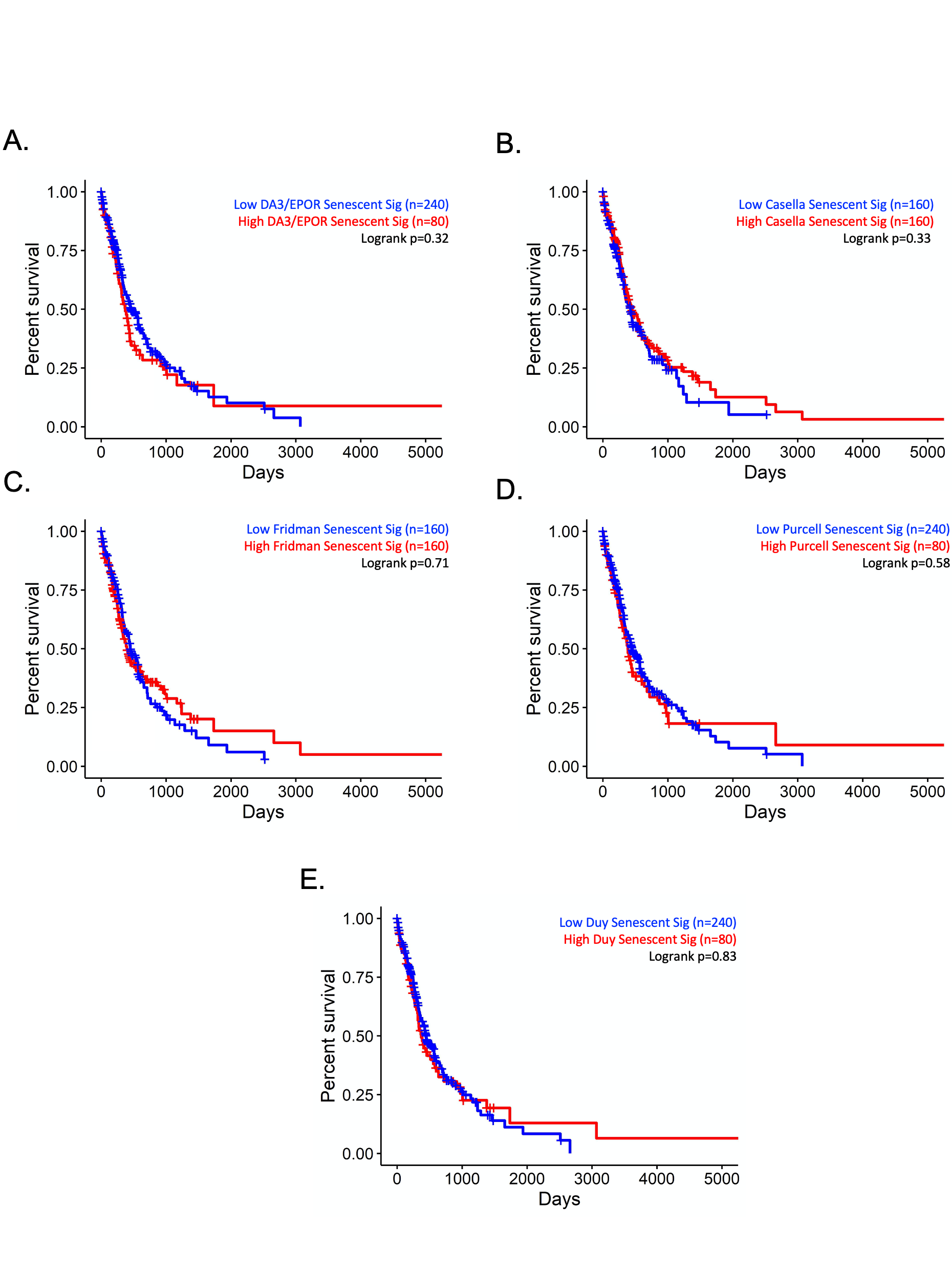

Supplement: Supplementary file 10 — Supplementary Figure 9 [file 41419_2023_6015_MOESM10_ESM.tif]

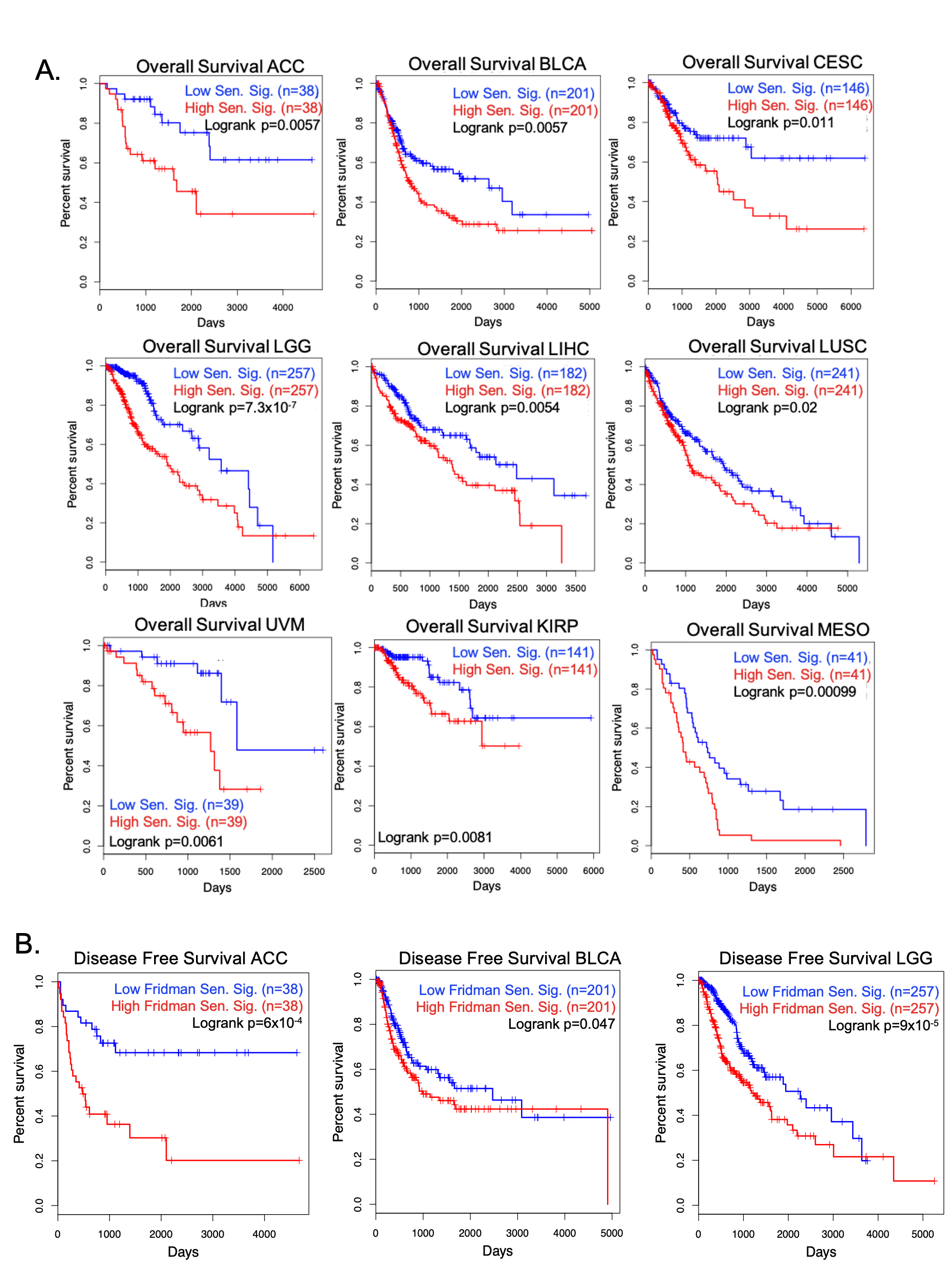

Supplement: Supplementary file 11 — Supplementary Figure 10 [file 41419_2023_6015_MOESM11_ESM.tif]

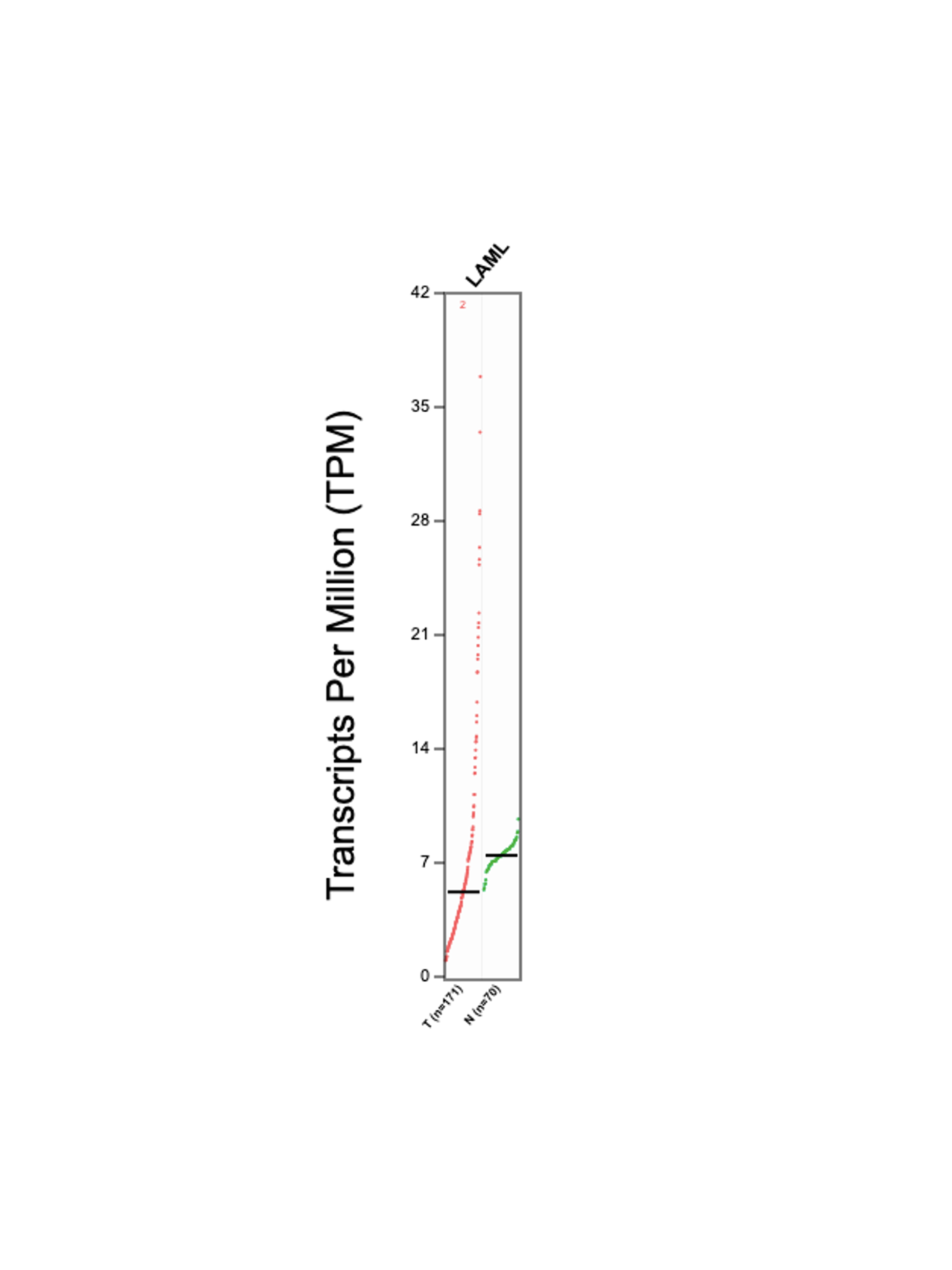

Supplement: Supplementary file 12 — Supplementary Figure 11 [file 41419_2023_6015_MOESM12_ESM.tif]
