# Supplementary material for: Heterogeneity in leukemia cells that escape drug-induced senescence-like state
Source: Cell Death Dis. 2023 Aug 5;14(8):503. doi: 10.1038/s41419-023-06015-4 (PMC10404232; doi:10.1038/s41419-023-06015-4)

Supplementary Figure 12.

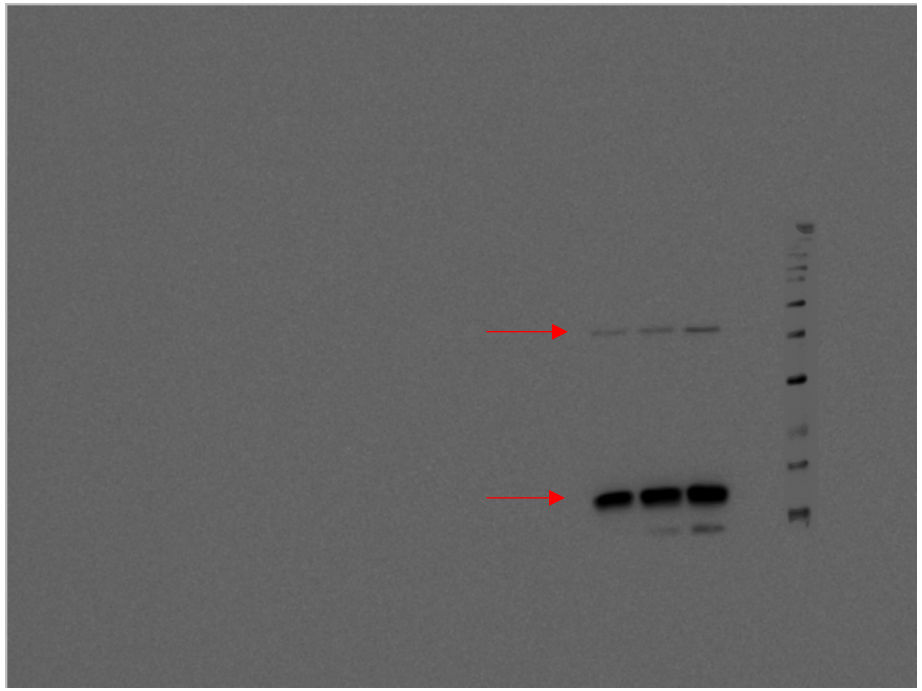

FL393 (p53)

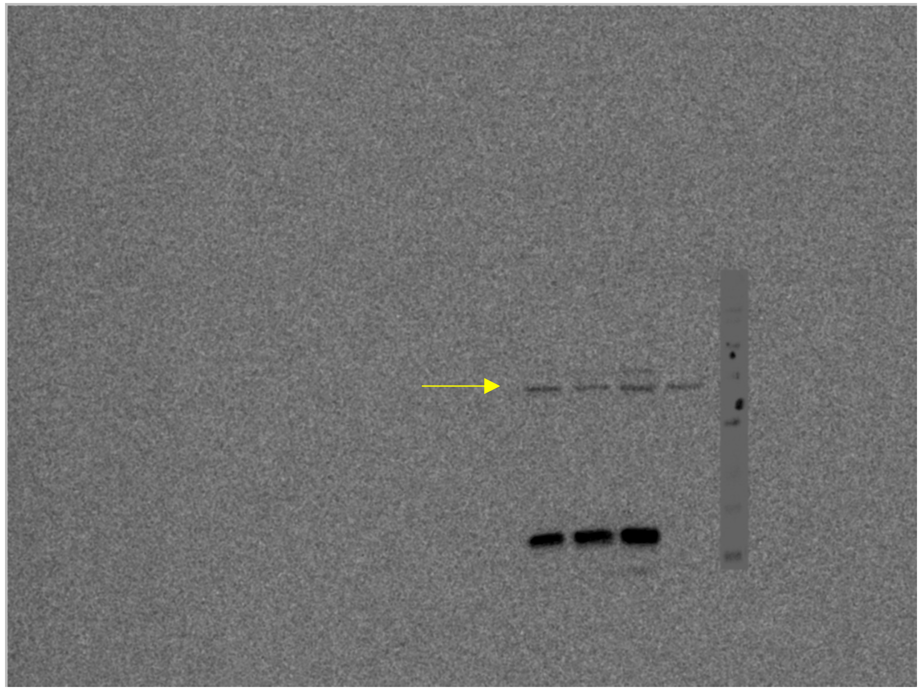

βactin

Supplementary Figure 13.

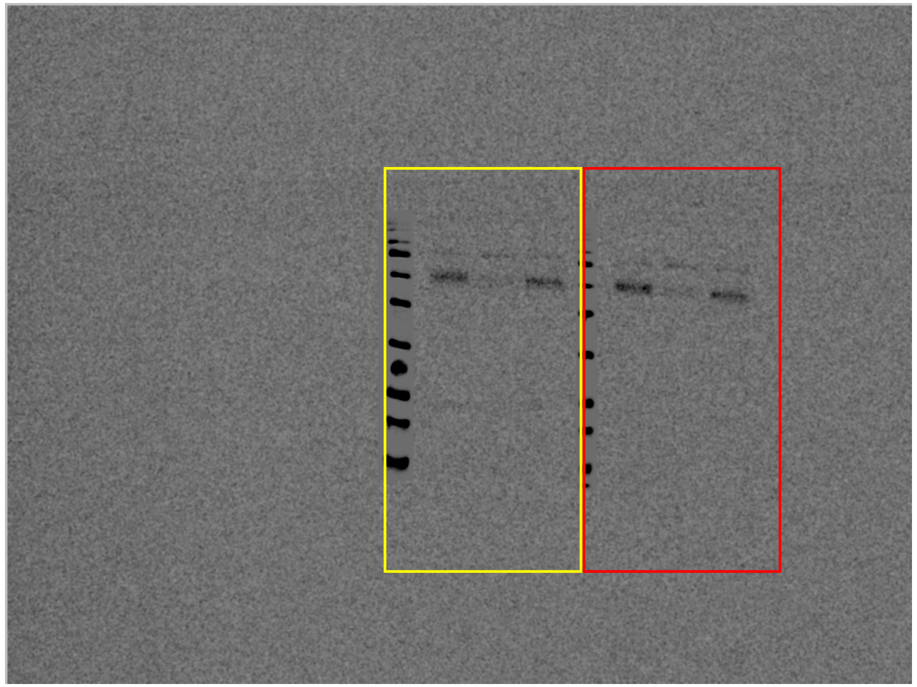

cMyc

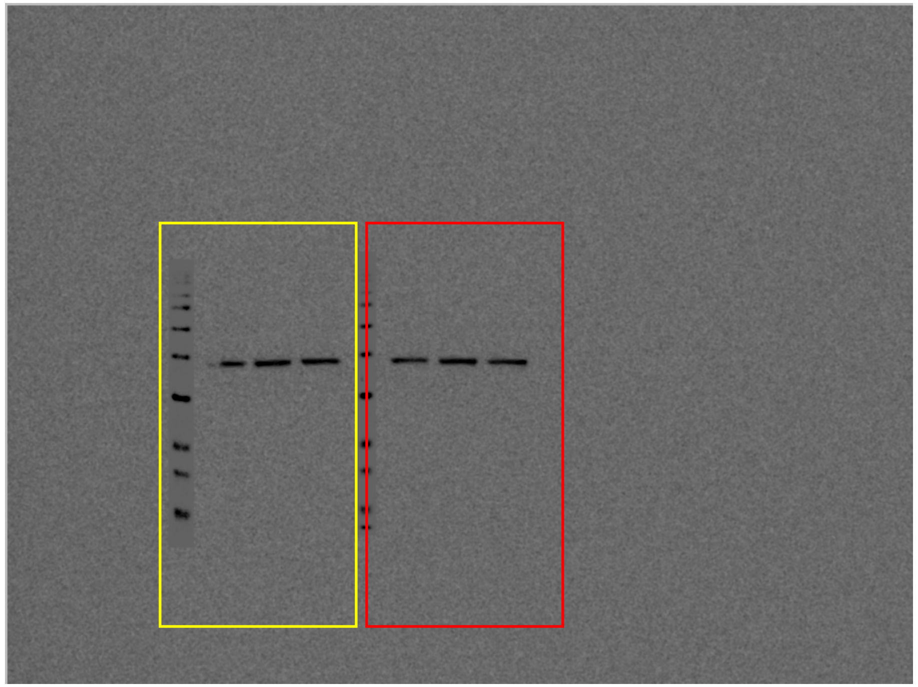

βactin

Supplementary Figure 14.

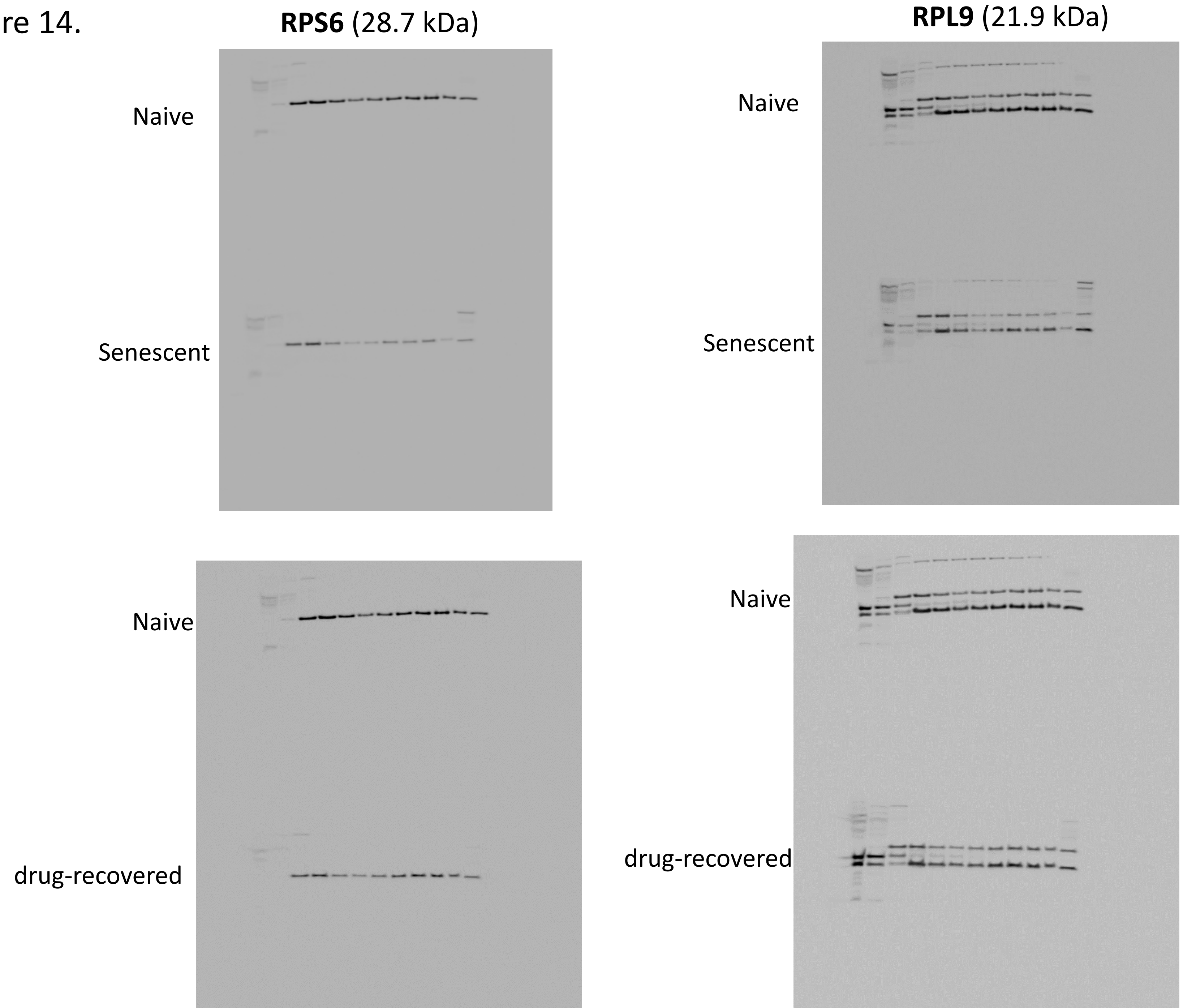

Supplement: Supplementary file 15 — Original data files [file 41419_2023_6015_MOESM15_ESM.pdf]
