# Supplementary material for: Heterogeneity in leukemia cells that escape drug-induced senescence-like state
Source: Cell Death Dis. 2023 Aug 5;14(8):503. doi: 10.1038/s41419-023-06015-4 (PMC10404232; doi:10.1038/s41419-023-06015-4)
Supplement: Supplementary file 16 — Supplementary information [file 41419_2023_6015_MOESM16_ESM.docx]

**Supplemental Information**

**Supplementary Methods**

**Cell Proliferation and Viability**

Total cells were counted on dates indicated on individual experiments. Cells were pelleted by centrifugation at 1000 rpm and resuspended in PBS. Cells were mixed with 1:1 mixture of PBS pH 7.4 and 0.4% trypan blue stain (Thermo). All viable cells were counted by hemocytometer; all 4 quadrants of the hemocytometer were counted and averaged. The number of live cells per ml was determined as: (average live cell number) x 2 x 10^5^.

**Drug Treatment**

DA3/EPOR cells were washed with phosphate buffer solution (PBS) followed by treatment with doxorubicin (Dox) (200 ng/ml, or at the specified concentrations), in the absence or presence EPO (1 U/ml). Human AML cell lines, OCIM2 and OCI-AML5, were treated with 200 ng/ml Dox or 0.1 μM or 0.25 μM Daunorubicin (DNR), respectively, for 24 hr. Chloroquine (CQ) was freshly prepared in sterile ddH2O prior to treating cells; Ammonium Chloride (NH4Cl) was solubilized in sterile ddH2O; sterile ddH2O served as a vehicle control for CQ and NH4Cl. Cells were treated with CQ and NH4Cl at the indicated concentrations for the desired times.

**SA-β-gal staining**

Briefly, cells were harvested and washed with PBS pH 7.4 followed by fixation in 3% formaldehyde for 20 min. Cells were washed with PBS, suspended and incubated overnight at 37°C in freshly made b-gal staining solution consisting of: 1 mg 5-bromo-4-chloro-3-indolyl P3-D-galactoside, 150 mM NaCl, 40 mM citric acid pH 6.0, 40 mM sodium phosphate pH 6.0, 5 mM potassium ferrocyanide, 5 mM potassium ferricyanide, and 2 mM MgCl2.

**Cell cycle analysis by Flow cytometry**

Cells were pelleted by centrifugation and washed with PBS and fixed with cold 75% ethanol before storing at -20°C until use. Fixed cells were pelleted and washed with PBS and suspended in in Staining Buffer (0.2% Triton X-100, 1 mM ethylenediaminetetraacetic acid [EDTA], in PBS) followed by incubation with RNaseA (100 μg/ml, ThermoScientific) at 37°C for 30 min. Cells were stained with 50 μg/ml propidium iodide (PI) for 1 hr. PI fluorescence was measured using a BD FACSCalibur flow cytometer (Becton Dickinson). PI fluorescence, cell size and granularity, and data analysis was performed using the BD CellQuest Pro software. Cell size and granularity was measured based on forward scatter and side scatter.

**LysoTracker**

LysoTracker Red DND-99 was used according to the manufacturer’s instructions. Briefly, 1x LysoTracker Red DND-99 was incubated with live cells for 30 min at 37°C with 5% CO2. Cells were pelleted, washed with PBS and incubated with DAPI for 10 min. Cells were resuspended in ProLong Gold anti-fade medium and placed on cover slides and allowed to cure overnight.

**Cell size**

Cells were mixed at a 1:1 ratio with 0.4% trypan blue and pl 46 aced on a hemocytometer. Multiple random fields of views were imaged, and pixels were manually calculated on ImageJ. Pixel lengths were converted to μm by calculating the amount of pixels of a known reference length. Trypan blue positive (dead) cells and cells with irregular (non-spherical) shapes were excluded from the analysis.

**Quantitative real-time PCR (qRT-PCR)**

Cells were pelleted and stored at -80°C until use. Total RNA was isolated by TRIzol (Life Technologies) according to the manufacturer’s instructions. RNA was solubilized in RNA cleaning buffer consisting of: 75% ethanol, 75 mM NaOAc, 50 ug/ml glycogen, and stored at -80°C for 1 hr. RNA was pelleted, washed with 75% ethanol and resuspended in nuclease free H2O. RNA concentration and purity was measured using a NanoDrop One spectrophotometer (Thermo Scientific). cDNA synthesis was performed with 2 μg of total RNA using the High Capacity RNA-to-cDNA Kit (ThermoFisher Scientific) and subjected to PCR using the SsoFaster EvaGreen Supermix Kit (BioRad) on a DNA Engine Opticon 2 Real-Time PCR Detector (BioRad). Samples were run in technical triplicate. Relative expression was calculated using the 2−ΔΔCt method; Ct values were normalized to mouse Rps18 or human GAPDH. Primer efficiencies were determined for all previously unpublished primers prior to use in qRT-PCR. Statistical analysis was performed on the ΔΔCt values.

The following oligonucleotides were used as qRT-PCR primers:

Mouse:

C3 F: 5’AAG CAT CAA CAC ACC CAA CA

C3 R: 5’CTT GAG CTC CAT TCG TGA CA

ICAM1 F: 5’GGTCCTTGCCTACTTGCTG

ICAM1 R: 5’CTGTGCTTTGAGAACTGTGG

ITGB2 F: 5’GTCCTTCCGACAGTTTCTCTC

ITGB2 R: 5’GGAGTCATGGAGTGTGGTATC

P16 F: 5’AAT CTC CGC GAG GAA AGC

P16 R: 5’GTC TGC AGC GGA CTC CAT

Rps18 F: 5’GTG TTG AGG AAA GCA GAC AT

Rps18 R: 5′CAG TCT GGG ATC TTG TAC TG

SerpinB2 F: 5’CAC CAC AGG GGG ATT TG

SerpinB2 R: 5’ACC CTT CGG GTA GCA GGT TT

vWF F: 5’CAG CAT CTC TGT GGT CCT GA

vWF R: 5’GAT GTT GTT GTG GCA AGT GG

Human:

C3 F: 5’AGTCTCCTGCTTTAGTGATGC

C3 R: 5’GCCTTTGTTCTCATCTCGCT

GAPDH F: 5’ GGA GCG AGA TCC CTC CAA AAT

GAPDH R: 5’ GGC TGT TGT CAT ACT TCT CAT GG

SerpinB2 F: 5’- GTT CAT GCA GAT CCA GA

SerpinB2 R: 5’- CGC AGA CTT CTC ACC AAA CA

vWF F: 5’ACTCACACAAAGTCTTCTCACA

vWF R: 5’GCTCATGCAACATCTCCTCTG

**RNA-seq analysis**

DA3/EPOR cells were harvested and frozen at -80°C, 3- or 9-days after Dox treatment (24 hr); untreated control cells were harvested on the 3rd day along with the treated samples. Total RNA was isolated using Monarch Total RNA Miniprep Kit (New England BioLabs) as described by the manufacturer. RNA was isolated for all samples on the same day. RNA-seq was performed in biological triplicate by Genome Quebec, using the Illumina NovaSeq 6000 at a depth of 25 million pair-ended reads that were 100 bp in length. Genome Quebec performed quality control and ensured that all samples had a >6.8 RIN value prior to performing a poly-A tail selection and converting the RNA into cDNA. RNA-seq alignment was performed on an Amazon EC2

instance Linux terminal. Although the Quality Control (QC) did not detect any illumina adapter sequences, the first 13 bp were trimmed using Flexbar. HiSAT2 was then used to align reads to the MM10 indexed genome. HTSEQ was used to generate read counts, which were then used for differential expression. Differential expression was performed on Rstudio using EdgeR. Gene Set Enrichment Analysis (GSEA) was used to investigate differentially regulated pathways and gene sets. GSEA used EdgeR normalized read counts. Rstudio was used to generate heatmaps and perform principal component analysis (PCA). RNA expression data was generated using GEPIA2 using publicly available data from TCGA and GTEx.

**Western Blotting and Antibodies**

Cells were pelleted and washed with PBS. Cells were then lysed in 1 x SDS (sodium dodecyl sulfate) lysis buffer (1% SDS, 10% glycerol, 88 mM Tris- HCl pH 6.8, in water) and boiled at 97°C for 10 min. A Pierce BCA Protein assay (Cat#23225) was used to measure protein

concentration of lysates according to the manufacturer’s instructions. Once protein

concentrations were measured, 0.1% bromophenol blue and 0.1M dithiothreitol (DTT) was

added to the lysed sample. 50 μg of total protein lysate per sample was separated by SDS

polyacrylamide gel electrophoresis and transferred onto nitrocellulose membranes. Membranes

were blocked for 1 hr at room temperature (RT) with PBST blocking buffer (5% milk in PBS and 0.1% Tween 20), followed by an overnight incubation with primary antibodies diluted in blocking buffer at 4°C. The following primary antibodies were used: FL393 (sc-6243, Santa Cruz Biotechnology), RPL9 (Abcam, ab182556), RPS6 (Abcam, ab40820), c-Myc (Santa Cruz Biotechnology, sc-764) and β-actin (Santa Cruz Biotechnology, sc-47778). Membranes were washed with PBST and incubated for 1 hr at RT in HRP secondary antibody diluted in blocking buffer. Membranes were washed with PBST and incubated with Pierce ECL Western blotting substrate for 1 min. Western blots were imaged on a MicroChemi System (DNR Bio Imaging System) and analyzed on a GelCaptureChemi program or using film.

**Polysome Analysis**

Polysome profiling was performed by fractionation as described (<https://bio-protocol.org/e833>). Briefly, DA3/EPOR cells were incubated with 100 μg/mL cycloheximide for 5 minutes prior to lysis. Cells were washed twice in PBS containing 100 μg/mL cycloheximide and lysed in

hypotonic lysis buffer (5 mM Tris-HCl (pH 7.5), 2.5 mM MgCl2, 1.5 mM KCl, 1X Halt™ & Protease Inhibitor Cocktail (ThermoFisher Scientific, catalogue number 78425), 100 μg/mL

cycloheximide, 2 mM DTT, 200 Units/mL SUPERase In™ RNase Inhibitor (ThermoFisher

Scientific, catalogue number AM2696), 0.5% (v/v) Triton X-100 and 0.5% (w/v) sodium

deoxycholate). Cell lysates were prepared by centrifugation for 5 minutes at 20,000 g at 4°C and 20 A260nm was loaded onto a 7-step sucrose gradient prepared in sucrose buffer (20 mM

HEPES (pH 7.6), 100 mM KCl, 5 mM MgCl2 and 100 μg/mL cycloheximide) followed by

centrifugation at 30,000 rpm for 3 hr in a Beckman SW41 rotor at 4°C (acceleration: max., deceleration: no brake). The absorbance was measured at 254 nm in a continuous flow and

fractions were collected. Protein samples were precipitated in 10% trichloroacetic acid overnight at -20°C. The protein pellet was collected by centrifugation for 10 minutes at 10,000 g at 4°C, followed by two acetone washes and resuspension in 2X Laemmli buffer. Protein samples were separated by SDS-PAGE and immunoblotted.
